# Organoid-based neutralization assays reveal a distinctive profile of SARS-CoV-2 antibodies and recapitulate the real-world efficacy

**DOI:** 10.1073/pnas.2509616122

**Published:** 2025-08-28

**Authors:** Zhixin Wan, Cun Li, Ying Zhou, Yifei Yu, Man Chun Chiu, Jingjing Huang, Shuxin Zhang, Xiaoxin Zhu, Qiaoshuai Lan, Yanlin Deng, Wei Xue, Chengfan Jiang, Jiali Wu, Zijun Zhao, Jian-Piao Cai, Lin Huang, Yong Zhang, Xiaojuan Liu, Zheng Zhang, Hin Chu, Linqi Zhang, Zhiwei Chen, Kelvin Kai-Wang To, Kwok-Yung Yuen, Hans Clevers, Jie Zhou

**Affiliations:** ^a^Department of Microbiology, Li Ka Shing Faculty of Medicine, The University of Hong Kong, Pokfulam, Hong Kong, China; ^b^Centre for Virology, Vaccinology and Therapeutics, Hong Kong Science and Technology Park, Hong Kong, China; ^c^BiomOrgan Ltd., Hong Kong, China; ^d^Clinical Stem Cell Research Center, Peking University Third Hospital, Beijing 100191, China; ^e^Institute for Hepatology, National Clinical Research Center for Infectious Disease, Shenzhen Third People’s Hospital, Shenzhen 518112, China; ^f^State Key Laboratory of Emerging Infectious Diseases, The University of Hong Kong, Hong Kong, China; ^g^Pandemic Research Alliance Unit at the University of Hong Kong, Hong Kong, China; ^h^Center for Global Health and Infectious Diseases, Comprehensive Acquired Immunodeficiency Syndrome Research Center and Beijing Advanced Innovation Center for Structural Biology, School of Medicine, and Vanke School of Public Health, Tsinghua University, Beijing 100084, China; ^i^Oncode Institute, Hubrecht Institute, Royal Netherlands Academy of Arts and Sciences, and University Medical Center Utrecht, Utrecht 3584 CT, the Netherlands; ^j^Roche Pharmaceutical Research and Early Development, Basel CH-4070, Switzerland

**Keywords:** organoid, neutralizing antibody, SARS-CoV-2

## Abstract

The efficacy of VIR-7831, a class 3 anti-SARS-CoV-2 monoclonal antibody (mAb), was demonstrated repeatedly in clinical trials; yet, reduced neutralization against Omicron variants in cell-line-based neutralization assays led to its withdrawal from clinical use. We developed organoid-based neutralization assays to measure mAb potency. We found that most class 3 mAbs, especially those not blocking receptor-binding domain-ACE2 binding, including VIR-7831, were substantially underestimated in cell-line-based assays. Nasal organoids adequately recapitulated the real-world effectiveness of VIR-7831 because of biologically relevant low ACE2 expression, and exclusively reproduced the in vivo protection of S2 mAbs due to the high TMPRSS2 expression, reminiscent of native human respiratory epithelial cells. Collectively, the robust organoid culture system and biologically relevant expression profiles of ACE2 and TMPRSS2 make nasal organoids present a correlate of in vivo protection of neutralizing mAbs exclusively. The organoid-based neutralization assays, superior to conventional cell-line-based assays, can recapitulate and predict the real-world efficacy of mAbs.

## Significance

VIR-7831, a class 3 anti-SARS-CoV-2 mAb with verified clinical effectiveness, was withdrawn since it showed diminished potency against Omicron variants in cell-line-based neutralization assays. The underestimated potency of VIR-7831 in conventional neutralization assays prompted us to establish organoid-based neutralization assays and hypothesize that organoid-based assays could provide a better in vitro correlate of protection and recapitulate real-world scenarios of antibody effectiveness. We demonstrated that many class 3 mAbs, particularly those not blocking receptor-binding domain (RBD)-ACE2 binding, were substantially undervalued in cell-line-based neutralization assays. Nasal organoids adequately recapitulated the real-world efficacy of VIR-7831 and in vivo protection of S2 mAbs since nasal organoids, a biologically relevant model, retain the cellular gene expression profile intrinsic to native human respiratory epithelial cells.

Severe acute respiratory syndrome coronavirus 2 (SARS-CoV-2), a betacoronavirus closely related to the human SARS-CoV-1, has evolved and developed escape mutations since late 2020. Vaccines, antiviral drugs, and monoclonal antibodies (mAbs) have been developed at an unprecedented speed and magnitude, greatly reducing transmission and disease severity in COVID-19 patients. Multiple virus-targeting mAbs have been approved for clinical use in treating clinically vulnerable and high-risk individuals. These antibodies are designed to reduce COVID-19 severity by binding to the SARS-CoV-2 spike glycoprotein, preventing the virus from binding to the ACE2 receptor on the surface of human cells. Since August 2023, EG.5 of the Omicron XBB sublineage became dominant in multiple countries with increased prevalence and immune evasion, and subsequently replaced by JN.1 (1), followed by KP.2 and KP.3 (2).

We and others have developed an array of virus-targeting mAbs against SARS-CoV-2 to treat high-risk COVID-19 patients (3–5). Based on structures of distinct neutralizing antibodies binding to SARS-CoV-2 spike trimer or receptor-binding domain (RBD), Barnes et al. classified mAbs into 4 classes (6). Class 1 and 2 antibodies directly block RBD-ACE2 binding, while class 3 antibodies target outside the ACE2 binding site. Some class 3 antibodies block RBD-ACE2 binding, while others do not (7). Unfortunately, the potency of many neutralizing antibodies, particularly of most class 1 and class 2 mAbs, has been largely compromised due to the emergence of immuno-evasive variants (8). Neutralization assays in immortalized cell lines (Vero E6, 293T-ACE2, Huh-7, etc.) are considered the “gold standard” for in vitro testing the potency of antibodies and antisera against SARS-CoV-2 infection. These assays measure the concentration of mAbs required to prevent infection of target cells by an authentic virus, or more commonly, a pseudovirus carrying the spike protein of wild-type SARS-CoV-2 (WT) or SARS-CoV-2 variants. However, substantial variations exist in these cell lines and different assay protocols (9). As such, the measurement of inhibitory concentrations (IC50, IC90) using these cell lines has been highly variable. Fold reduction in neutralization (FRN) was calculated to represent the resistance of a variant to a mAb relative to its ability to neutralize the wild-type virus; thus, FRN is a comparative reading, rather than an objective measurement of neutralizing potency. There have been concerns regarding the rationale of identifying potent mAbs, and even approving or withdrawing a mAb for clinical use, based on these in vitro neutralization assays (10).

Cell line-based neutralization assays have rapidly developed and contributed tremendously to the development of mAbs and vaccines. However, the ultimate effect of SARS-CoV-2 mAbs and vaccination sera is to neutralize the virus and protect human respiratory cells from infection, especially the epithelial cells in the upper airways, the primary targets of SARS-CoV-2 and many other respiratory viruses. Cell lines are convenient laboratory tools, yet they typically consist of a homogeneous population of transformed cells. Thus, cell lines can not recapitulate the human respiratory epithelium, which is composed of multiple cell populations with distinct morphology and diverse physiological functions. Mounting evidence has demonstrated that cell lines differ fundamentally from human respiratory cells in the context of complex interaction with SARS-CoV-2, including viral entry pathways (11) and usage of proviral host proteases (12). As such, cell-line-based neutralization assays might be unable to present a correlate of in vivo protection and reveal the real-life scenario of antibody effectiveness.

A major discrepancy has emerged between the gold standard neutralization assay and real-world efficacy data. The parent antibody S309 of VIR-7831 (also known as sotrovimab) was identified in memory B cells of a recovered SARS patient. This class 3 mAb, like other class 3 mAbs such as BD55-3152 (8), targets an epitope in the spike receptor binding domain that does not compete with ACE2 binding (13). In a recent WHO living guideline on therapeutics and COVID-19, the rationales for issuing VIR-7831 the emergency use authorization (EUA) for the treatment of high-risk COVID-19 patients and subsequent withdrawal of the EUA were specified (14). In brief, after a single infusion of 500 mg, the mean serum concentration was 117.6 µg/mL at 1 h and 24.5 µg/mL on day 29. Upon correction for 6.5 to 12% antibody penetration from serum to lung (as described for other mAbs), VIR-7831 concentration in the lung at day 29 after infusion would be 1.59 to 2.94 µg/mL. As measured in Vero E6 cells, IC90 to ancestral strain USA WA1/2020 was 0.19 µg/mL, around 10-fold lower than the lung antibody concentration at day 29, which was sufficient to neutralize USA WA1/2020 in COVID-19 patients. However, IC90 to BA.2 omicron was 25.3- and 48.1-fold higher than to preomicron variants (14). As such, it would be unlikely to achieve the concentration for sufficiently neutralizing BA.2 in the lung. In addition, “the above analysis was relevant to many Omicron sublineages including BA.4, BA.5, and BQ.1 etc.” as claimed in the living guideline. Thus, the US FDA withdrew the EUA of VIR-7831 in April 2022. WHO issued a “strong recommendation for the treatment of COVID-19 patients” with the antibody in September 2022 (14). However, UK and EU health authorities continued to use VIR-7831 for high-risk COVID-19 patients given the absence of approved alternatives (10). Interestingly, in November 2022, a large-scale clinical cohort study reported that VIR-7831 outperformed molnupiravir in preventing COVID-19 hospitalization and death during BA.2 wave (15). The real-world effectiveness of VIR-7831 during BA.2 predominance was verified in clinical studies worldwide (16, 17) and animal experiments (18–20). Thus, the “gold standard” neutralization assay misjudged the efficacy of VIR-7831; an effective mAb was available and still benefiting the patients, but was not offered to them when they were at risk of progressing to severe COVID-19.

The S2 stem helix region is a promising target to block SARS-CoV-2 infection broadly (21). Several S2 stem helix-specific neutralizing antibodies were isolated from recovered COVID-19 patients (22). Intriguingly, in contrast to the substantial protection in animal experiments, these S2 antibodies showed low neutralizing potency in conventional cell-line-based neutralization assays (23, 24). In addition, a poor correlation between in vitro measurement of neutralizing activity and in vivo protection was repeatedly reported in some classes of mAbs against influenza and Ebola viruses (25, 26). Thus, there is an unmet demand for a biologically relevant neutralization assay, a correlate of in vivo protection, that can accurately define the mAb neutralization potency to protect human respiratory cells (rather than immortalized cell lines) from viral infections and inform real-world efficacy.

We have previously established a respiratory organoid culture system directly from the primary lung tissues of patients upon surgical resection. The derived organoids are stably expanded for at least half a year. In the 2-phase organoid culture system, the expansion medium enables the initial organoid derivation and long-term expansion by directing the organoids toward an immature state; and differentiation protocols trigger maturation and generate mature airway and alveolar organoids that faithfully simulate the native airway epithelium and alveolar epithelium, respectively (27–30). Nasal organoids are derived using nasal epithelial cells procured from volunteered donors noninvasively; they are also long-term expandable, like the organoids from primary lung tissues. Upon induction of maturation, the differentiated nasal organoids contain four major airway epithelial cell types, similar to airway organoids; yet they retain the attributes of the upper airway epithelium (31, 32). All differentiated respiratory organoid models, including nasal, airway, and alveolar organoids, faithfully simulate the multicellular composition of the native respiratory epithelium and their related physiological functions, including the mucociliary escalator of human airways. Thus, the respiratory organoid culture system enables us to rebuild and propagate the entire human respiratory epithelium in culture plates with excellent efficiency and stability. These physiologically active respiratory organoids have become robust and optimal tools for studying SARS-CoV-2 and other respiratory viruses (27–33). The misjudged efficacy of VIR-7831 in cell line-based neutralization assays prompted us to establish organoid-based neutralization assays with the hypothesis that organoid-based neutralization assays could provide a better in vitro correlate of protection and recapitulate real-world scenarios of antibody effectiveness against SARS-CoV-2 infection. We found that most class 3 antibodies, especially those not blocking RBD-ACE2 binding, such as VIR-7831, were underestimated substantially in most cell lines expressing a high level of ACE2, in contrast to nasal organoids and native human respiratory cells with paradoxically low ACE2 level. Moreover, nasal organoids exclusively reproduced the in vivo potency of S2 mAbs due to the biologically relevant high TMPRSS2 expression in nasal organoids and the usage of TMPRSS2-mediated viral entry of SARS-CoV-2 and SARS-CoV-1. Thus, organoid-based neutralization assays can objectively recapitulate and predict the real-world efficacy of mAbs.

## Results

### Characterizing Nasal Organoids of 96-Transwell Format and Organoid Infections of Coronaviruses.

**Fig. 1.**
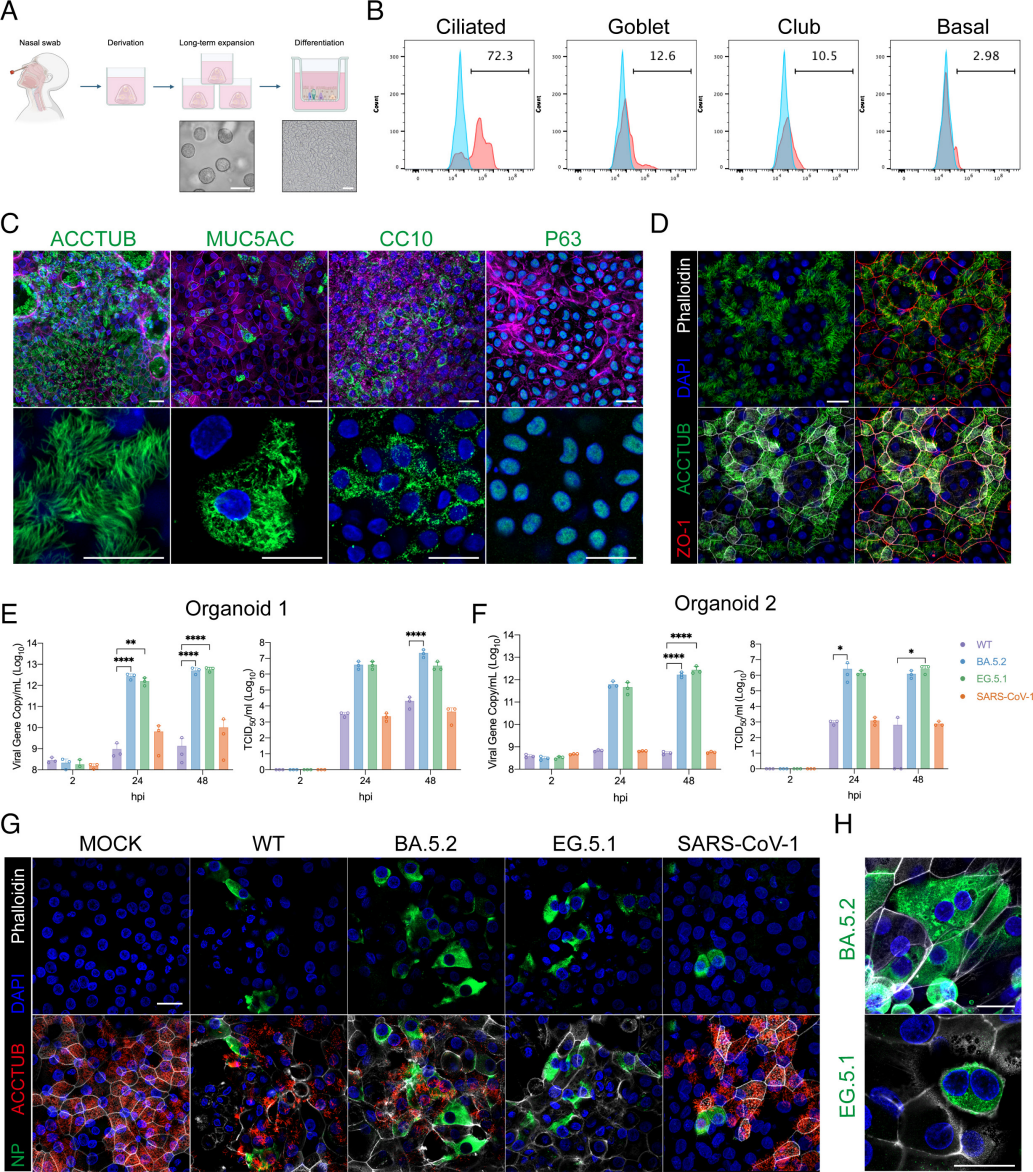
Characterizing nasal organoids of 96-transwell format and coronavirus infections. (*A*) A schematic graph outlines human nasal organoid derivation, expansion, and differentiation, including the derivation of nasal organoids from nasal cells, the expansion of undifferentiated nasal organoids in a 3D format, and the differentiation culture of nasal organoid monolayers onto 96-transwell inserts. A photomicrograph of the 3D undifferentiated organoids on day 6 and that of differentiated organoid monolayers on day 14 are shown underneath the corresponding stages. (Scale bar, 50 μm.) The schematic graph is created with BioRender. (*B*) Flow cytometry was performed to quantify the abundance of ciliated (ACCTUB^+^), goblet (MUC5AC^+^), club (CC10^+^), and basal (P63^+^) cells in the differentiated nasal organoid monolayers. Representative histograms show the percentage of cells labeled with cell-type-specific antibodies (red) and those labeled with isotype controls (blue). (*C*) Confocal images of ACCTUB^+^ ciliated cell, MUC5AC^+^ goblet cell, CC10^+^ club cell, and P63^+^ basal cell in differentiated nasal organoids. Nuclei and actin filaments were counterstained with DAPI (blue) and Phalloidin-647 (Purple), respectively. (Scale bar, 20 μm.) (*D*) Nasal organoids were costained for ciliated cells (ACCTUB, green), tight junctions (ZO-1, red), and F-actin filaments (Phalloidin-647, white). Nuclei were counterstained with DAPI (blue). (Scale bar, 20 µm.) (*E* and *F*) Nasal organoids were inoculated with the indicated SARS-CoV-1 and SARS-CoV-2 variants. Viral release from the apical side of nasal organoid monolayers was measured by RT-qPCR (*Left*) and TCID50 (*Right*) at the indicated hpi in organoids derived from two donors. Data represent means ± SD of three biological replicates. Two-way ANOVA with Tukey’s multiple comparison test was used for statistical analysis. *P < 0.05, **P < 0.01, ****P < 0.0001. (*G*) Immunofluorescent images of infected cells in nasal organoids after inoculation of the indicated virus; viral nucleoprotein (NP, green), ciliated cells (ACCTUB, red), DAPI (blue), and Phalloidin-647 (white). (Scale bar, 20 μm.) (*H*) Immunofluorescent images of syncytial bodies in nasal organoids infected by the indicated viruses. At 24 h after inoculation (MOI, 0.5), the organoid monolayers were fixed and costained for viral nucleoprotein (NP, green), F-actin filaments (Phalloidin-647, white), and nuclei (DAPI, blue). (Scale bar, 20 μm.)

As aforementioned, we have established nasal organoids using nasal cells collected noninvasively from the nasal mucosa of volunteered donors (illustrated in Fig. 1*A*). The undifferentiated organoids can be stably passaged for half a year. Upon induction of maturation, the differentiated nasal organoids faithfully mimic the upper respiratory epithelium better than airway organoids, although they accommodate the same cell populations (32). Based on our previous observations, when seeded on 24- or 12-transwell inserts to induce differentiation, nasal and airway organoids were more mature and sustained more robust viral growth, compared to the differentiated organoids of 3D formats (30, 32). In this study, we elected to use nasal organoid monolayers on 96-transwell inserts, in a similar format to immortalized cells seeded in 96-well plates in conventional neutralization assays. First, we demonstrated that the differentiated nasal organoids grown on 96-transwell inserts (termed nasal organoids thereafter) achieved adequate maturation to mimic the upper respiratory nasal epithelium, akin to those grown on the larger transwell inserts. The differentiated nasal organoids accommodate ample ciliated cells, a dominant cell population in the native nasal epithelium. The other 3 airway epithelial cell types, goblet, club, and basal cells, are also present in the nasal organoids (Fig. 1 *B* and *C*). Zonula occludens-1 (ZO-1, a tight-junction associated protein) is distributed in the organoid monolayer in close association with F-actin filaments (Fig. 1*D*), indicating the formation of epithelial integrity, an essential host defense mechanism of the human respiratory epithelium.

Repetitive outbreaks of zoonotic coronaviruses in the past decades and constant mutation of SARS-CoV-2 call for the development of broad-spectrum neutralizing antibodies. Indeed, several very effective SARS-CoV-2 mAbs have been isolated from peripheral blood mononuclear cells of SARS-CoV-1 convalescent patients (3, 34). Before testing the neutralizing activity of mAbs against these coronaviruses, we profiled the replication of SARS-CoV-1, as it has not been interrogated in respiratory organoids so far. We inoculated 3 strains of SARS-CoV-2 (WT, BA.5.2, and EG.5.1) together with SARS-CoV-1 in the nasal organoids to observe viral replication kinetics. All these coronaviruses replicated in nasal organoids. BA.5.2 and EG.5.1 of the Omicron lineage replicated more actively than the WT strain. SARS-CoV-1 exhibited a comparable replicative fitness to SARS-CoV-2 WT (Fig. 1*E*). The higher replicative fitness of BA.5.2 and EG.5.1 than that of SARS-CoV-2 ancestral strain WT and SARS-CoV-1 was reproduced in nasal organoids derived from another donor (Fig. 1*F*). The active replication of Omicron BA.5.2 and EG.5.1, and the less robust propagation of SARS-CoV-1 and SARS-CoV-2 WT were verified in immunostaining and confocal imaging (Fig. 1*G*). Syncytial bodies were present in EG.5.1 and BA.5.2 infected nasal organoids (Fig. 1*H*), in line with our previous findings (31).

### Fabricating Nasal Organoid-Based Neutralization Assays.

**Fig. 2.**
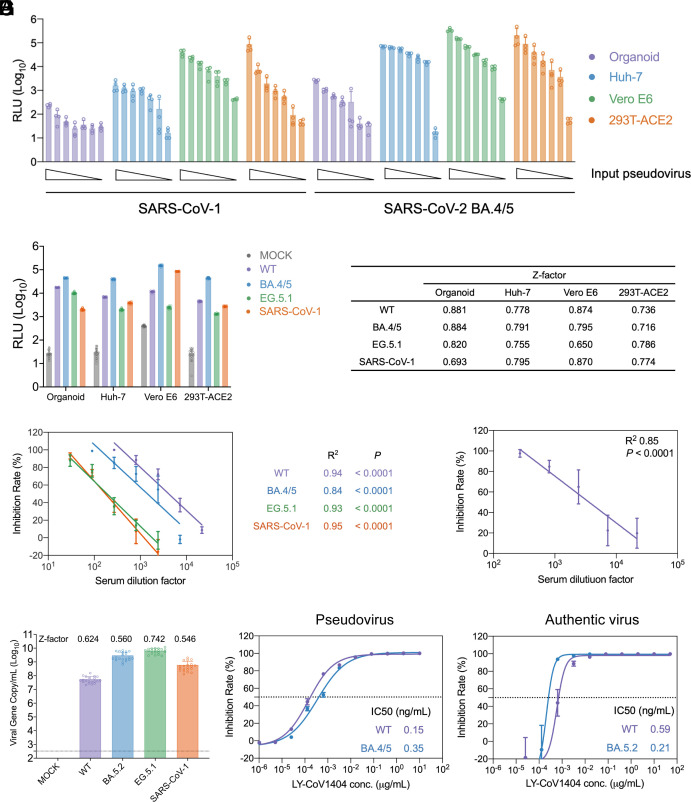
Fabricating nasal organoid-based neutralization assay and assessing mAb potency. (*A*) Nasal organoids and the indicated cell lines were inoculated with decreasing doses of SARS-CoV-1 and SARS-CoV-2 BA.4/5 pseudoviruses. At 72 h postinoculation, cell lysates were harvested for luciferase assay to assess pseudovirus entry. The results show the relative luciferase activity (relative light unit) of the indicated pseudovirus versus pseudovirus particles without carrying an envelope protein. Data represent the means ± SD in a representative experiment (n = 4) independently performed twice. (*B*) Signal window and Z-factor of the indicated pseudovirus infection in the indicated models. The *Left* panel presents the means ± SD in a representative experiment (n = 20). (*C* and *D*) The neutralization potency of a vaccination serum of SARS-CoV-2 WT was tested against the indicated pseudovirus entry in nasal organoids. Simple linear regression analysis was used to assess the correlation between inhibition and dilution factor. (*C*) Nasal organoids derived from one donor were applied to test the neutralization activity of the vaccination serum against the indicated pseudoviruses; (*D*) Nasal organoids derived from three different donors were applied to evaluate the serum neutralization against SARS-CoV-2 WT pseudovirus. (*E*) Viral RNA released from nasal organoids 24 hpi of SARS-CoV-1 (1 MOI) or SARS-CoV-2 variants (0.01 MOI) and Z-factor values (n = 20). (*F* and *G*) The neutralization curves of LY-CoV1404 and IC50s to the indicated pseudoviruses (*F*) and (*G*) authentic viruses. The horizontal dotted lines indicate a 50% neutralization. Data represent means ± SD (n = 4).

To establish a robust organoid-based pseudovirus assay for measuring the neutralization potency of mAbs, we first examined SARS-CoV-1 and BA.4/5 pseudovirus entry in nasal organoids, and three commonly used cell lines for neutralization assays, Huh-7, Vero E6, and 293T-ACE2 in parallel. Upon inoculation with serially diluted pseudoviruses, BA.4/5 pseudovirus showed a higher entry efficiency than SARS-CoV-1 pseudovirus in the organoids and three cell lines (Fig. 2*A*), lending support to the higher replication capacity of BA.5 than SARS-CoV-1 (shown in Fig. 1 *E* and *F*). Moreover, the entry efficiency of BA.4/5 and SARS-CoV-1 pseudoviruses was substantially higher in three cell lines than in nasal organoids. This is conceivable as nasal organoids, an in vitro model of the native upper airway epithelium, have all the cellular defense mechanisms to protect from microbial invasion, e.g., the motile cilia, and mucin secreted by goblet cells, which are absent in immortalized cell lines. Given the distinct entry efficiency in cell lines and organoids, we optimized pseudovirus infection of WT, BA.4/5, EG.5.1, and SARS-CoV-1 in organoids and cell lines, including the amount of inoculum and incubation time. To obtain a greater signal window in the organoids, we used a greater amount of inoculum (around 100-fold higher) in the organoids than in the cell lines. We settled on an optimized setting for each model; the signal window and Z-factor are shown in Fig. 2*B*.

We then validated the organoid-based neutralization assay by testing a COVID-19 vaccination serum in nasal organoids against the four pseudoviruses. The serum was collected from a volunteer vaccinee who received three doses of the Sinovac inactivated vaccine of SARS-CoV-2 ancestral strain and one dose of mRNA‐based vaccine BNT162b2 of an ancestral spike. The vaccination serum showed a dose-dependent inhibition against WT, BA.4/5, EG.5.1, and SARS-CoV-1 pseudovirus with R^2^ of 0.94, 0.84, 0.93, and 0.95, respectively (Fig. 2*C*). The escalating immune escape of Omicron variants BA.4/5 and EG.5.1 was revealed in the organoid-based neutralization assay. We also tested the serially diluted antiserum against WT pseudovirus in nasal organoids derived from three different donors. The dose-dependent inhibition was verified (Fig. 2*D*). We observed a high correlation between inhibition and dilution factors among the organoids from these 3 donors (R^2^ 0.85; *P* < 0.0001). Namely, the potential interindividual variations among organoids from different donors barely affected the measurement of neutralizing potency, thus validating the reproducibility and robustness of the organoid-based neutralization assay.

We next set up an organoid authentic virus neutralization assay to validate the measurements of the pseudovirus neutralization assay. As aforementioned, nasal organoids were less susceptible to SARS-CoV-1 than to SARS-CoV-2. To optimize the experimental conditions, we employed a high MOI (1.0) inoculation for SARS-CoV-1 and a low MOI (0.01) inoculation for SARS-CoV-2 (including WT, BA.5.2, and EG.5.1) and examined viral gene copy number 24 hours postinfection (hpi). A satisfactory signal window and Z-factor for each virus strain were obtained under the optimized setting (Fig. 2*E*). We also tested the neutralizing activity of VIR-7831 against SARS-CoV-2 WT and BA.5.2 infection in nasal organoids via immunostaining of SARS-CoV-2 nucleocapsid protein, followed by imaging analysis. VIR-7831 neutralized the two virus strains in a dose-dependent manner, with a stronger inhibitory effect in SARS-CoV-2 WT than in BA.5.2 (*SI Appendix*, Fig. S1). We next tested a class 3 antibody, LY-CoV1404, in pseudovirus infection using organoids derived from a donor and authentic virus infection using organoids from another donor, to assess the correlation between the two assays. Notably, the 50% inhibition concentrations (IC50) obtained from two distinct (pseudovirus and authentic virus) neutralization experiments using nasal organoids from different donors were highly consistent (Fig. 2 *F* and *G*). Namely, the organoids from different donors applied to pseudovirus and live virus assays yielded a consistent neutralization profile. Here, the pseudovirus neutralization assay measures the entry efficiency of the replication-defective virus in a single infection cycle in the presence or absence of mAbs, whereas authentic virus neutralization assays detect viral growth after multiple virus life cycles. Although the readouts from the two assays showed more or less discrepancy in this study and others (13), the general neutralization profile and trend remained consistent. Nonetheless, both assays should be performed to complement and validate each other. Overall, we established robust organoid-based pseudovirus and authentic virus neutralization assays that presented consistent results in the context of the potential interindividual variations among organoids from different donors, thus allowing us to reliably measure antibody neutralization potency against infections of SARS-CoV-2 and SARS-CoV-1.

### Distinct Neutralization Profile of VIR-7831 Revealed in Organoids and Cell Lines.

**Fig. 3.**
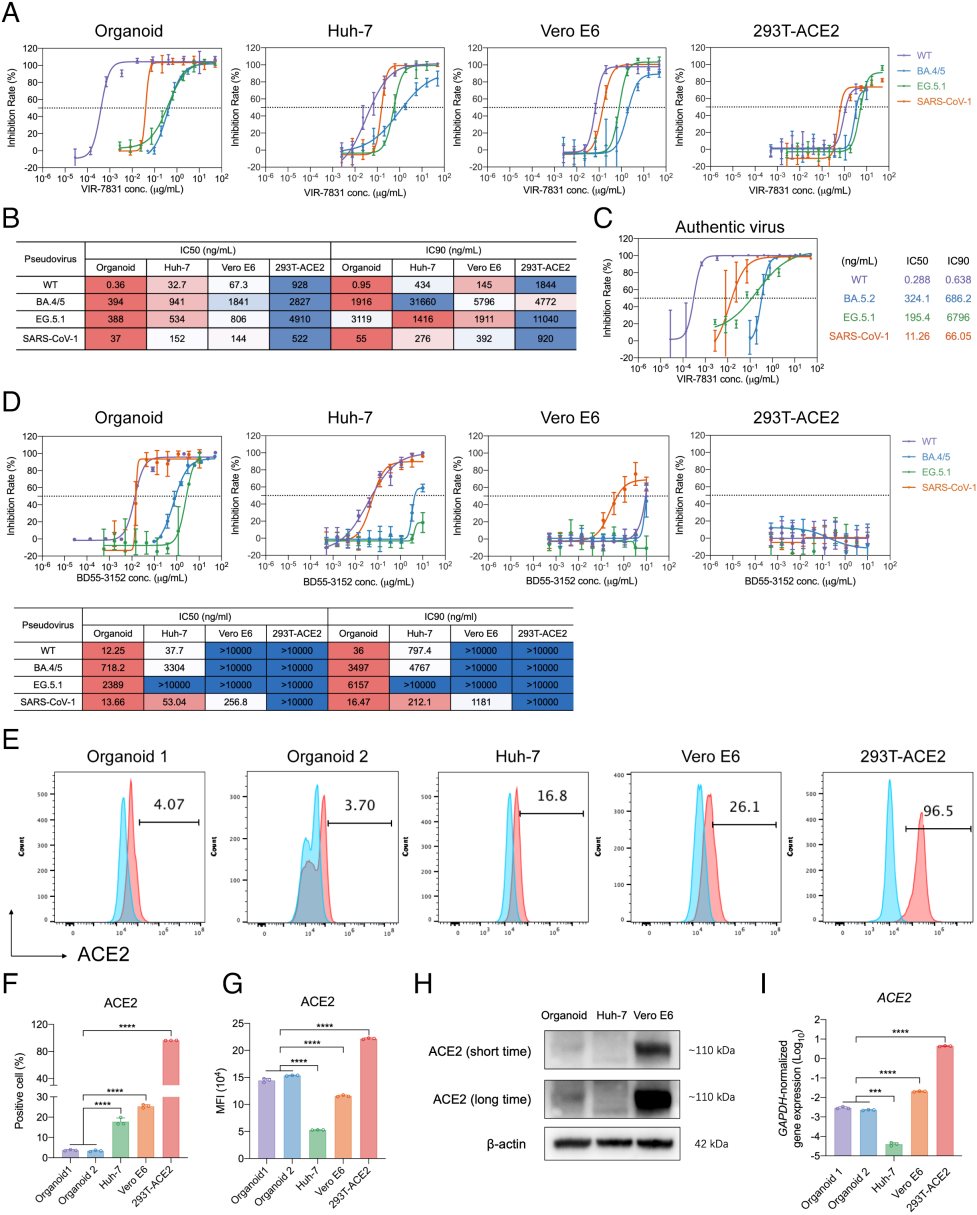
Distinct neutralization profile of VIR-7831 related to the ACE2 expression level. (*A*) Neutralization curves of VIR-7831 against the indicated pseudoviruses in organoids and the cell lines. Data show the mean ± SD (n = 4). (*B*) The heatmap shows the IC50 and IC90 values (ng/mL) of VIR-7831 against the indicated pseudoviruses. The neutralizing potency is red-white-blue color-coded, with red being the strongest neutralization of each mAb to the indicated pseudoviruses among the test models. (*C*) The neutralization curve of VIR-7831 against the indicated authentic viruses in nasal organoids. The horizontal dotted line indicates 50% neutralization. Data are mean ± SD (n = 4). (*D*) Neutralization activity of BD55-3152 against the indicated pseudoviruses in organoids and the cell lines. Data show the mean ± SD (n = 4). The heatmap shows the IC50 and IC90 values (ng/mL) of BD55-3152 against the indicated pseudoviruses. The neutralizing potency is red-white-blue color-coded, with red being the strongest neutralization of each mAb to the indicated pseudoviruses among the test models. (*E–G*) ACE2 expression in two lines of organoids and the indicated cell lines was analyzed by flow cytometry. (*E*) Representative histograms show the percentage of ACE2^+^ cells (red) in the organoids and cell lines. Isotype control (blue). (*F*) The percentage of ACE2^+^ cells and (*G*) MFI in the organoids and cell lines. Data represent the means ± SD of a representative experiment (n = 3). Two-tailed Student’s *t* test. ****P < 0.0001. (*H*) A representative western blot of short and long exposure reveals ACE2 expression in organoids and indicated cell lines. The experiment was independently performed three times. (*I*) GAPDH normalized ACE2 mRNA expression in two lines of organoids and cell lines. Data are presented as mean ± SD (n = 3) of a representative experiment independently performed three times. Two-tailed Student’s *t* test. ***P < 0.001, ****P < 0.0001.

As aforementioned, the real-world effectiveness of VIR-7831 was not reproduced in standard cell-line-based neutralization assays. To reveal the neutralization profile of VIR-7831 in the organoid-based assays and evaluate whether the organoid-based assays reveal a correlate of in vivo protection, more accurately than standard cell-line-based neutralization assays, we measured the IC50 and IC90 of VIR-7831 against SARS-CoV-2 WT, BA.4/5, EG.5.1, and SARS-CoV-1 in nasal organoids, Huh-7, Vero E6, and 293T-ACE2 cells in parallel (Fig. 3 *A* and *B*). First, the IC50 and IC90 to the WT pseudovirus were 0.36 ng/mL and 0.95 ng/mL respectively in organoids. As mentioned above, we inoculated the nasal organoids with a 100-fold higher dose of pseudovirus than the cell lines. The low IC50 and IC90 (<1 ng/mL) to WT pseudovirus in nasal organoids highlighted the superior potency of VIR-7831, in contrast to its low neutralization potency previously shown in cell lines (35, 36). Second, a dramatically reduced neutralizing potency against BA.4/5 and EG.5.1 relative to WT was revealed in organoids. Nonetheless, IC50s to Omicron variants remained in a low range (<1 µg/mL). Third, VIR-7831 showed strong neutralizing activity against SARS-CoV-1 pseudovirus (IC50 37 ng/mL). Moreover, the neutralization profile revealed in pseudovirus infection was verified in authentic virus infections (Fig. 3*C*); and a high consistency (R^2^ 0.892; r 0.944) of two assays (*SI Appendix*, Fig. S2*A*) was noted as shown in LY-CoV1404 (Fig. 2 *F* and *G*). We repeated the experiment in pseudovirus and live virus infection in nasal organoids derived from other donors and obtained consistent results (*SI Appendix*, Fig. S3).

As aforementioned, the recent WHO living guideline on therapeutics and COVID-19 clarified the rationales to issue the EUA for VIR-7831 and its subsequent withdrawal (14). VIR-7831 IC90 to ancestral strain USA WA1/2020 in Vero E6 cells was 0.19 µg/mL, 8.4- to 15.5-fold lower than the lung antibody concentration at day 29, which would be 1.59 to 2.94 µg/mL upon correction with the serum-to-lung penetration rate. Yet IC90 to BA.2 omicron was 25.3- and 48.1-fold higher than to preomicron variants (14), indicating it would be unlikely to achieve the concentration for sufficient neutralization of BA.2 in the lung. This constituted the rationale for the FDA to withdraw the EUA for VIR-7831. However, based on the measurements of organoid-based pseudovirus assays, IC90 to BA.4/5 (1.916 µg/mL) was far (12.8-fold) lower than the serum concentration at day 29 (24.5 µg/mL) postadministration. The protection would be even more prominent if IC90 in live BA.5.2 infection (686 ng/mL) is used for the projection, since the serum antibody concentration at day 29 (24.5 µg/mL) exceeded the IC90 by 35.7-fold.

In Huh-7 cells (Fig. 3 *A* and *B*), IC50 against WT SARS-CoV-2 (32.7 ng/mL) was 91-fold higher than that in the organoids (0.36 ng/mL). Yet the IC50 to BA.4/5 (941 ng/mL) and EG.5.1 (534 ng/mL) in Huh-7 cells were comparable to those in organoids (BA.4/5, 394 ng/mL; EG.5.1, 388 ng/mL). Despite an IC50 of 941 ng/mL against BA.4/5 in Huh-7 cells, the peak inhibition was around 80%, indicating a compromised neutralization in the cell line. In contrast, VIR-7831 reached 100% inhibition with an IC50 of 394 ng/mL in the organoids. Vero E6 cells revealed a similar neutralization profile to Huh-7 cells, with slightly higher IC50s, which accurately replicated the results documented in the WHO living guideline (14). The paralleled assay in 293T-ACE2 cells exhibited a very distinct profile; the IC50s and IC90s to all tested pseudoviruses were substantially higher than those in organoids, Huh-7, and Vero E6 cells (Fig. 3 *A* and *B*). Despite an IC50 of 522 ng/mL to SARS-CoV-1, the maximum inhibition was 80%. Overall, the weak neutralization of VIR-7831 in all the tested cell lines fully supported the rationale leading to its withdrawal as documented in the WHO living guideline. Moreover, its weak neutralization to all the pseudoviruses in 293T-ACE2 cells was consistent with prior findings that the potency of class 3 antibodies has been heavily underestimated in ACE2 high-expression cell lines (9, 18).

Another class 3 antibody BD55-3152, which is also isolated from the memory B cells of a convalescent SARS patient and targets the same region as VIR-7831 (8), showed a very similar neutralization profile to VIR-7831 in nasal organoids. The distinctive neutralization profile of VIR-7831 displayed in organoids and cell lines was also reproduced when BD55-3152 was tested (Fig. 3*D*), verifying that VIR-7831 was indeed underestimated in conventional cell-line-based neutralization assays. Collectively, parallel assays indicated that the class 3 antibody VIR-7831 revealed distinct neutralizing potency in organoids and three cell lines. Compared with organoids, Huh-7, and Vero E6 cells, especially 293T-ACE2 cells, underrated the potency of VIR-7831 against all the interrogated coronaviruses. Despite the dramatically reduced potency, VIR-7831 retained substantial neutralizing activity against Omicron variants BA.5.2 and EG.5.1 as revealed in organoid-based neutralization assays. Namely, nasal organoids adequately and exclusively recapitulated the clinically verified effectiveness of class 3 antibody VIR-7831, among all the interrogated in vitro models.

### ACE2 Expression Underlying the Distinct Neutralization Profile of Antibodies in Organoids and Cell Lines.

Prior studies reported that the potency of non-ACE2 competing antibodies (including class 3 antibodies) was substantially underestimated in ACE2 high-expression cell lines (9, 18). We inferred that ACE2 expression levels in organoids and the cell lines may underlie the distinct measurements of VIR-7831’s neutralization potency. As such, we examined ACE2 expression in the cell lines (Huh-7, Vero E6, and 293T-ACE2) and two lines of nasal organoids derived from two different donors by flow cytometry (Fig. 3*E*). In the 293T-ACE2 cell line, almost all cells highly expressed ACE2, as expected. The percentage of ACE2^+^ cells was significantly lower in nasal organoids than in Vero E6 and Huh-7 cells (Fig. 3*F*). Despite a small portion of cells in nasal organoids expressing ACE2, the mean fluorescence intensity (MFI) of ACE2^+^ cells in the organoids was significantly higher than that of Huh-7 and Vero E6 cells (Fig. 3*G*). Nonetheless, Western blot showed that ACE2 was highly expressed in Vero E6 cells, exceedingly more abundant than in Huh-7 cells and nasal organoids (Fig. 3*H*), which was verified by confocal imaging (*SI Appendix*, Fig. S4) and the abundance of mRNA transcripts (Fig. 3*I*). Of note, ACE2 expression level in organoids, Vero E6, and 293T-ACE2 cells negatively correlated to VIR-7831’s neutralization potency manifested in these models (Fig. 3*B* and *SI Appendix*, Fig. S5). The paradoxically low ACE2 expression in the native human airway cells, the primary target of SARS-CoV-2, has been amply evidenced in single-cell sequencing analysis (37), transcriptomic and protein profiling (38). Huh-7 cells were chosen deliberately for the comparison due to the low ACE2 level (39). Nonetheless, Huh-7 cells displayed a neutralization profile distinct from that in nasal organoids; thus low ACE2 alone did not enable Huh-7 cells to present a correlate of in vivo protection. Taken together, disparate neutralization profiles of VIR-7831 in nasal organoids and cell lines suggested that antibody neutralization and protection from virus infections in human respiratory cells are poorly recapitulated in cell lines, while biologically relevant nasal organoids represent a more optimal tool for assessing mAb neutralization potency.

### Higher Neutralizing Potency of Class 3 Antibodies than Class 1 Antibodies Revealed in Organoids.

**Fig. 4.**
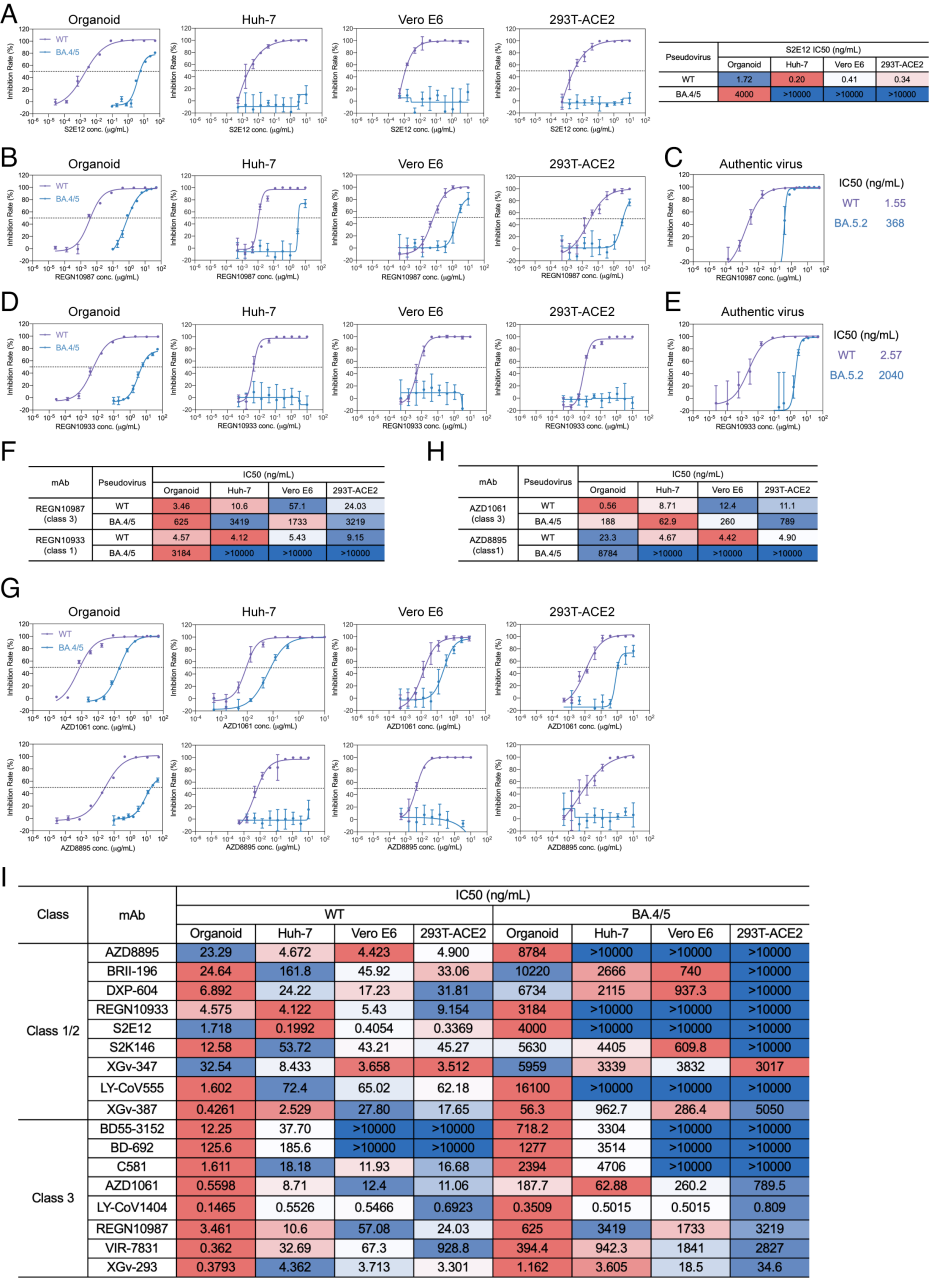
Higher neutralizing potency of class 3 antibodies than class 1 antibodies in organoids. (*A*) The neutralization curve and heatmap of S2E12 against indicated pseudoviruses in organoids and cell lines. (*B*–*F*) The neutralization curve and heatmap of REGN10987 and REGN10933. (*G* and *H*) AZD1061 and AZD8895 against indicated pseudoviruses or authentic viruses in organoids and cell lines. The horizontal dotted lines indicate 50% neutralization. Data are presented as mean ± SD (n = 4). (*I*) Neutralization activity of mAbs of class 1/2 and class 3 in different models against SARS-CoV-2 WT and BA.4/5 pseudoviruses. The neutralizing activity is red-white-blue color-coded, with red being the strongest neutralization of each mAb among the test models.

While the ACE2 expression level in target cells substantially impacted the measured neutralizing potency of class 3 antibodies, it barely affected the measured potency of class 1 antibodies (9, 18). Most commonly used cell lines for neutralization assays, except Huh-7, highly express ACE2, particularly those engineered cell lines overexpressing ACE2. We inferred that the relative neutralization potency of class 1 and class 3 mAbs might be misjudged in high ACE2-expression cell lines, and the potency of other class 3 antibodies might be generally underestimated in cell-line-based neutralization assays. We measured the activity of an “ultrapotent” class 1 antibody S2E12 (36) in the organoid pseudovirus neutralization assay (Fig. 4*A*). Its IC50 to WT pseudovirus (1.72 ng/mL) was around fivefold higher than that of VIR-7831 (0.363 ng/mL). We also tested S2E12 in Huh-7, Vero E6, and 293T-ACE2 cells (Fig. 4*A*). The IC50s lower than 1 ng/mL against the WT pseudovirus in all three cell lines reproduced its “ultrapotency” as reported previously (35, 36). Consistent with the prior reports, S2E12, like most class 1 antibodies, lost neutralization activity against Omicron BA.4/5. In nasal organoids, S2E12 neutralized BA.4/5 pseudovirus (IC50, 4,000 ng/mL, peak inhibition around 80%) much less efficiently than VIR-7831 (394 ng/mL). A dramatically reduced potency of class 1/2 antibodies LY-CoV555 and BRII-196 to Omicron was reproduced in the organoid-based neutralization assay (*SI Appendix*, Fig. S6). Overall, organoid-based neutralization assay revealed a higher potency of class 3 antibody VIR-7831 to SARS-CoV-2 viruses than class 1 antibody S2E12, since high ACE2 expression in the cell lines distorted the neutralization profile.

Ronapreve, a cocktail of two mAbs REGN10933 (a class 1 antibody, casirivimab) and REGN10987 (a class 3 antibody, imdevimab), targets two different sites on the RBD of SARS-CoV-2 spike protein. Its use led to an over 80% relative risk reduction in developing symptomatic COVID-19 infections and a significant reduction in patient mortality in hospitals (40). Due to the reduced potency against Omicron variants in cell-line-based neutralization assays (41), the US FDA withdrew the EUA in early 2022 (14). The IC50 of REGN10987 to WT pseudovirus was 3.46 ng/mL in organoids (Fig. 4 *B* and *F*); the IC50 to BA.4/5 pseudovirus was elevated to 625 ng/mL. In organoid infection of the live BA.5.2 virus, the IC50 was 368 ng/mL (Fig. 4*C*), suggesting the class 3 mAb retained its potency against the Omicron virus. However, the class 1 antibody REGN10933 had an IC50 of 3,184 ng/mL and 2,040 ng/mL to BA.4/5 pseudovirus and BA.5.2 live virus, respectively (Fig. 4 *D*–*F*). Thus, organoid-based assays demonstrated that the class 3 antibody REGN10987 was more potent and more resistant to Omicron’s immune evasion than the class 1 antibody REGN10933, similar to the comparative neutralization potency of VIR-7831 and S2E12.

Similar to VIR-7831, the potency of the class 3 REGN10987 to WT and BA.4/5 pseudovirus was higher in organoids than in three cell lines, with a peak inhibition of 100% in organoids versus around 80% in cell lines. Again, cell line-based neutralization assays underrated its potency and exaggerated the resistance of the Omicron variant to REGN10987. Intriguingly, unlike S2E12, which was overrated in cell lines, the class 1 REGN10933 neutralized WT pseudovirus in organoids, Huh-7, Vero E6, and 293T-ACE2 at a comparable level. Nonetheless, REGN10933 lost its neutralization potency to BA.4/5 both in organoids and the cell lines, similar to most class 1 counterparts.

Evusheld, a combination of two long-acting antibodies AZD8895 (a class 1 antibody) and AZD1061 (a class 3 antibody) developed by AstraZeneca, was authorized for the prevention and treatment of COVID-19. Based on the result of a phase III clinical trial, Evusheld significantly reduced the relative risk of progressing to severe COVID-19 or death (42). We examined the potency of the two antibodies against WT and BA.4/5 pseudoviruses in organoids and cell lines (Fig. 4 *G* and *H*). The organoid-based neutralization assay verified the higher potency of class 3 AZD1061 than that of class 1 AZD8895. Except for Huh-7, the potency of AZD1061 to WT and BA.4/5 was higher in organoids than in the two cell lines. Similarly, class 1 AZD8895 showed a higher potency to WT pseudovirus in all 3 cell lines than in organoids, and it lost neutralization activity to BA.4/5 in cell lines and organoids.

Apart from the 3 pairs of class 3 and class 1 antibodies shown above, we tested more class 3 and class 1/2 antibodies in organoids and three cell lines in parallel and observed a diverse neutralization profile in different models (Fig. 4*I*). In fact, the neutralization profile of class 1/2 antibodies varied in organoids and cell lines. Some antibodies, including S2E12, AZD8895, and XGv-347, showed a higher potency to WT pseudovirus in cell lines than in organoids, while others, such as S2K146, LY-CoV555, and XGv-387, displayed an opposite comparative profile in organoids and cell lines. Nonetheless, most class 1 mAbs were invariably vulnerable to immune evasion. Except for a class 1/2 antibody XGv-347 that showed low IC50s to BA.4/5 in organoids and cell lines, almost all the tested class 1/2 antibodies were vulnerable to BA.4/5 immune evasion.

Most class 3 antibodies retained substantial potency to BA.4/5 in organoids. However, not all class 3 antibodies were heavily underestimated in cell lines compared to organoids. As for other class 3 antibodies, including AZD1061, LY-CoV1404, and XGv-293, the underestimation was not as dramatic as that for VIR-7831 and BD55-3152. As aforementioned, class 3 antibodies varied in their capacity to block RBD-ACE2 binding. We noted that the class 3 antibodies with a low ACE2 competition level (8) were underestimated in cell lines more prominently, and vice versa (*SI Appendix*, Fig. S8). Namely, class 3 antibodies that do not compete for RBD-ACE2 binding (such as VIR-7831) were more dramatically underestimated in cell lines. Collectively, organoid-based assays revealed a higher potency of most class 3 antibodies than class 1 antibodies to neutralize SARS-CoV-2 viruses. Class 3 antibodies, especially those not competing for ACE2 binding, are generally underestimated in cell lines, particularly in high ACE2 expression cell lines; whereas the potency of some class 1 antibodies, but not all, is overestimated in cell-line-based neutralization assays.

### The In Vivo Protection of S2 Antibodies Exclusively Reproduced in Organoids.

Most SARS-CoV-2 mAbs are designed to block the S1 subunit of SARS-CoV-2 spike protein, especially RBD, from binding to the ACE2 receptor. However, constant mutations on the S1 subunit in SARS-CoV-2 variants abolished the neutralizing activities of these antibodies. In contrast, the S2 subunit is relatively conserved, especially across the beta-coronaviruses, making it a promising target for broadly neutralizing antibodies. The S1 subunit binding to the ACE2 receptor primes a secondary cleavage at the S2′ site by serine protease TMPRSS2 at the surface plasma membrane or cysteine protease cathepsins in the endosomal compartment. TMPRSS2 or cathepsin cleavage of the S2′ site exposes the concealed fusion peptide (FP); FP insertion in the host cell membrane triggers the conformational change of the S2 subunit from the prefusion state into the postfusion state, during which the S2 stem helix region becomes extended to form a stem-helix (43), and then induces the fusion of viral and cellular membrane, followed by viral genome release to initiate replication. It is expected that antibodies targeting the S2 stem helix region may block SARS-CoV-2 infection (21). Several S2 stem helix-specific neutralizing antibodies were isolated from recovered COVID-19 patients or generated via hybridoma technology, such as S2P6, CC40.8, and CV3-25 (22). Intriguingly, in contrast to the substantial protection in animal experiments, these S2 antibodies displayed low neutralizing potency in conventional cell-line-based neutralization assays (23, 24).

**Fig. 5.**
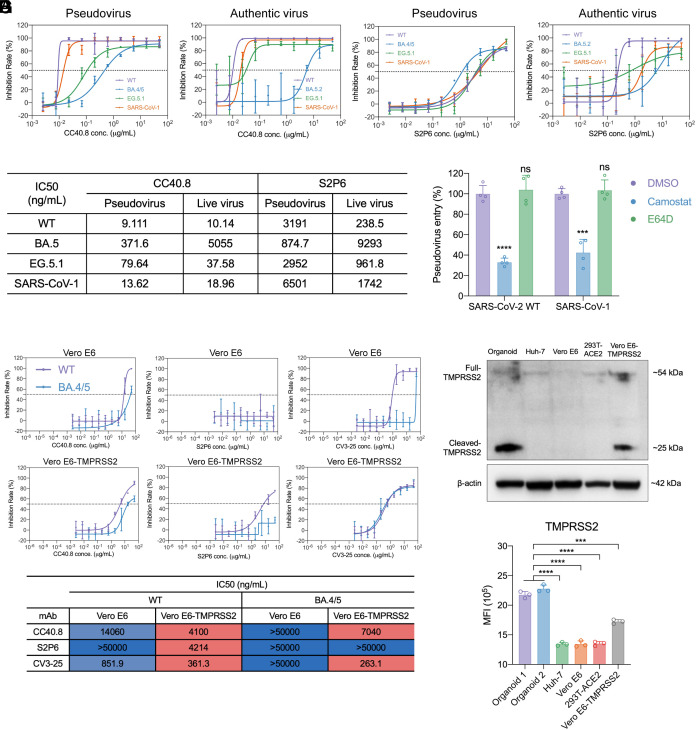
The in vivo protection of S2 antibodies was reproduced in organoid-based neutralization assays. The neutralization curve of (*A*) CC40.8, (*B*) S2P6, and (*C*) IC50 values against different pseudoviruses or live viruses in nasal organoids. Data are mean ± SD (n = 4). (*D*) The indicated pseudovirus entry into nasal organoids in the presence or absence of the serine protease inhibitors was measured by luciferase assay. The results show the relative luciferase activity of the inhibitor-treated organoids versus that of DMSO-treated organoids (n = 4). Data represent the mean ± SD of a representative experiment. Statistics were determined by one-way ANOVA with Tukey’s multiple comparisons test. ***P < 0.001, ****P < 0.0001. ns, not significant. (*E*) Neutralization curves and heatmap of three S2 mAbs against SARS-CoV-2 WT and BA.4/5 pseudoviruses in Vero E6 and Vero E6-TMPRSS2 cell lines. The neutralizing activity is red-blue color-coded, with red being the strongest of each mAb in different cell lines. (*F*) The representative western blot shows TMPRSS2 expression in organoids and indicated cell lines. Western blot was independently performed three times. (*G*) Flow cytometry analysis shows the MFI of TMPRSS2 in different models. Data represent the means ± SD of a representative experiment, n = 3. Two-tailed unpaired Student’s *t* test. ***P < 0.001, ****P < 0.0001.

Most cell lines (e.g., Vero, HEK293T, and A549) utilize endosomal protease cathepsins for SARS-CoV-2 cellular entry due to low TMPRSS2 level (11); yet mAbs do not readily access endosomes where the cathepsin-triggered S2 cleavage and conformational change occur. In contrast, nasal and airway organoids, similar to human respiratory cells, utilize TMPRSS2 abundantly expressed on the cell surface membrane (11, 31, 44), to which mAbs are readily accessible. As such, the usage of TMPRSS2-mediated cell surface entry in target cells is probably critical to demonstrate the potency of S2 antibodies. We hypothesized that nasal organoids might be able to reproduce the in vivo protection of these S2 antibodies, given the high cell surface TMPRSS2 in these organoids (30, 32). We tested CC40.8 and S2P6 in the organoid-based neutralization assays (Fig. 5 *A*–*C*). CC40.8 showed IC50s ranging from 9.1 to 371.6 ng/mL to SARS-CoV-2 and SARS-CoV-1 pseudoviruses, which were dramatically lower than the IC50s measured previously in Hela-ACE2 cells (24). Except for BA.5.2, the authentic virus assay verified its neutralization potency in the pseudovirus neutralization assay. The expected broad spectrum of CC40.8 was sufficiently verified in the live virus neutralization assay. S2P6 appeared less potent than CC40.8, yet it showed substantial breadth against all the tested pseudoviruses. Its broad spectrum and potency were reproduced in the live virus neutralization assay, except for BA.5.2. Overall, organoid-based neutralization assays revealed the potency of these S2 antibodies, which has been elusive in cell line-based neutralization assays.

We reported previously that the SARS-CoV-2 virus (WT and BA.4/5) predominantly used TMPRSS2 for cellular entry and viral replication in the airway and nasal organoids, recapitulating the dependency on TMPRSS2 in primary respiratory cells and experimental animals (31, 45, 46). We proceeded to assess whether SARS-CoV-1 utilized the same entry route by examining the spike-driven pseudovirus entry into nasal organoids in the presence or absence of Camostat (a TMPRSS2 inhibitor) and E64D (a cathepsin L/B inhibitor). SARS-CoV-2 WT spike-driven entry was abolished by Camostat (Fig. 5*D*), which was consistent with prior findings of ours and others. SARS-CoV-1 spike also utilized TMPRSS2 for cellular entry, as reported previously (47, 48), albeit to a slightly lesser extent than SARS-CoV-2 WT. E64D treatment did not affect the entry of SARS-CoV-2 and SARS-CoV-1 pseudoviruses. Thus, SARS-CoV-1 and SARS-CoV-2 viruses enter nasal organoids through the TMPRSS2-mediated surface membrane entry, lending support to the above finding that nasal organoids reproduce the in vivo potency of S2 antibodies due to the usage of TMPRSS2 for cellular entry.

To further verify the role of TMPRSS2 in the measured potency of S2 antibodies, we tested three S2 antibodies in Vero E6 and Vero E6-TMPRSS2 cell lines in parallel. The three interrogated S2 mAbs showed a higher potency in Vero E6-TMPRSS2 than in Vero E6 cells (Fig. 5*E*). Nonetheless, we noticed that the neutralization potency of CC40.8 and S2P6 was substantially lower in Vero E6-TMPRSS2 than in organoids. We then examined TMPRSS2 expression in Huh-7, Vero E6, Vero E6-TMPRSS2, 293T-ACE2 cells, and randomly selected nasal organoid lines. TMPRSS2 is expressed as a single-chain zymogen that undergoes autoproteolytic cleavage, leading to the liberation of the catalytically active protease domain of 25 kDa (49). Western blot showed more cleaved TMPRSS2 in nasal organoids than in Huh-7 and 293T-ACE2 cells (Fig. 5*F*). The cleaved TMPRSS2 was even more abundant in nasal organoids than in Vero E6-TMPRSS2. Flow cytometry analysis indicated that most cells in all the tested cell lines and organoids expressed TMPRSS2 (*SI Appendix*, Fig. S9). Yet, the MFI was significantly higher in organoids than in all the cell lines, including Vero E6-TMPRSS2 (Fig. 5*G*). Namely, despite the overexpression, the TMPRSS2 level in Vero E6-TMPRSS2 cells was lower than that in nasal organoids, which explained the higher potency of S2 antibodies in organoids than in Vero E6-TMPRSS2 cells.

## Discussion

Cell line-based neutralization assays have contributed significantly to the development of mAbs and vaccines against SARS-CoV-2. However, discrepancies have emerged between the measured potency of cell line-based neutralization assays and the in vivo protection of mAbs (10, 18, 20). Both cell line- and organoid-based neutralization assays measure the extent of pseudovirus entry and/or live virus infection into target cells in the presence of RBD-targeting mAbs. Conceivably, the expression of the ACE2 receptor mediating cellular entry of SARS-CoV-1 and SARS-CoV-2 is essential for the readout of the assays. We demonstrate that cell line-based assays exhibit a highly variable activity of mAbs since ACE2 is distributed disparately in these cell lines (Fig. 3), as reported previously (9, 18). Moreover, ACE2 expression in these cell lines is fundamentally distinct from that in native human respiratory cells (9, 11). Recognizing the ACE2 level on the target cells to be an important experimental variable for measuring the neutralization potency of mAbs, Farrell et al raised a critical question (9), “What target cell ACE2 expression provides the most biologically relevant measure of SARS-CoV-2 neutralization?.” From our viewpoint, target cells most biologically relevant to human airway epithelial cells can provide the most biologically relevant measure of SARS-CoV-2 neutralization.

Leveraging the robust respiratory organoid culture system established by our team and the high biological relevance of nasal organoids, we developed organoid-based neutralization assays to measure the neutralizing potency of mAbs against SARS-CoV-2 and SARS-CoV-1 (Fig. 2). These organoid-based assays deliver consistent neutralization profiles of mAbs, unaffected by potential variations among the organoids from different donors (Fig. 2 *D*, *F* and *G*). Due to the paradoxically low yet biologically relevant ACE2 expression, nasal organoids accurately recapitulate the real-world effectiveness of VIR-7831 (Fig. 3), which was seriously underrated in cell-line-based neutralization assays and led to the erroneous withdrawal of this effective mAb from clinical use. The organoid-based neutralization assays also revealed a high potency of most class 3 antibodies against SARS-CoV-2 Omicron variants, which has been generally underestimated in cell-line-based neutralization assays (Fig. 4). Moreover, the class 3 antibodies not competing for RBD-ACE2 binding (such as VIR-7831) were more dramatically underestimated in cell lines than those blocking ACE2 binding. Consistent with our finding (*SI Appendix*, Fig. S5), Lempp et al documented a negative correlation between the neutralization potency of non-ACE2-competing mAbs and ACE2 level on the target cells (18). The high ACE2 expression in most cell lines, which is not physiologically relevant to the native human airway epithelial cells (37), may distort the neutralization profile, contributing to the underestimated potency of non-ACE2-competing mAbs in these cell lines. The exact mechanism (s) leading to the underestimation is elusive, partially because the neutralization mechanisms of non-ACE2-competing mAbs remain poorly understood (9, 13). Yet multiple mechanisms have been proposed. Pinto reported that VIR-7831 neutralized the virus through several mechanisms, including S-glycoprotein trimer cross-linking, steric hindrance, and the aggregation of virions (13). The other two class 3 antibodies SP1-77 and 1G11 are also potent and broad neutralizing antibodies against SARS-CoV-2 variants. SP1-77, interacting with the N343 glycosylation site like VIR-7831, disrupted the fusion of viral and cellular membranes by preventing the dissociation of the S1 subunit of the spike protein (50); and 1G11 facilitated the cross-linking of spike proteins upon binding to the RBD, rather than blocking the RBD-ACE2 binding (51).

SARS-CoV-1 and SARS-CoV-2 preferentially use the cell surface TMPRSS2 to enter the native human respiratory cells (47, 48). Due to the high TMPRSS2 expression in nasal organoids and the usage of TMPRSS2-mediated cellular entry by these viruses, reminiscent of native human respiratory epithelial cells (11, 12), organoid-based neutralization assays reproduced the in vivo protection of S2 mAbs, which was not manifested in most cell lines due to low TMPRSS2 level, even in Vero E6-TMPRSS2 cells (Fig. 5). Overall, the robust organoid culture system and biologically relevant expression profile of ACE2 and TMPRSS2 give nasal organoids a unique edge to present a favorable correlate of in vivo protection of neutralizing mAbs, which is unachievable in commonly used cell lines. Therefore, organoids provide a robust preclinical model to assess the efficacy and safety of mAbs before advancing to human trials. Moreover, respiratory organoids hold great potential for developing antiviral drugs against respiratory viruses.

Apart from direct neutralization, mAbs may engage other protective mechanisms, such as Fc-dependent effector functions, including antibody-dependent cellular cytotoxicity, antibody-dependent phagocytosis, and complement activation, which are unable to be manifested in neutralization assays, including our organoid-based neutralization assays. In addition, nasal organoids indeed present a practical challenge; they do not grow as fast as cell lines. Nonetheless, the robust organoid culture system enables us to perform neutralization assays in 96-transwell plates, allowing medium-throughput testing sufficiently. As for high-throughput applications, including screening large amounts of mAbs, bioengineering techniques would be warranted. Taken together, the organoid-based neutralization assays recapitulate and predict the real-world efficacy of SARS-CoV-2 mAbs, superior to the conventional cell-line-based neutralization assays. More generally, our studies open an avenue to develop organoid-based neutralization assays for informing the effectiveness of mAbs and vaccination sera against other viruses. Lab-grown human organoids will be extensively leveraged in drug preclinical testing and accelerate drug development.

## Materials and Methods

### Establishment, Maintenance, and Differentiation of Nasal Organoids.

Nasal organoids were derived using nasal epithelial cells procured noninvasively from multiple healthy donors with a respiratory organoid expansion medium (BiomOrgan), upon the ethical approval of the Institutional Review Board at the University of Hong Kong/Hospital Authority Hong Kong West Cluster (UW21-695). All human samples used in this study were fully deidentified prior to experimentation, and informed consent was obtained from all donors. The derived organoids are consecutively passaged every 10 to 14 d for up to 6 mo, using the same expansion medium. The proximal differentiation to generate monolayers of mature nasal organoids on transwell inserts with differentiation medium (BiomOrgan) was performed as described previously (32). The differentiated nasal organoid monolayers grown on 96-transwell inserts were utilized throughout the study unless stated otherwise.

### Pseudovirus and Live Virus Neutralization Assays.

Neutralization assays were conducted by incubating pseudoviruses with serially diluted serum or purified mAbs at 37 °C for 1 h. Huh-7 cells, Vero E6, and 293T-ACE2 cells in 96-well plates, and nasal organoids in 96-well transwell plates were incubated with the virus–antibody mixture for 72 h and then applied to luciferase assays. Two controls are included in each plate: a virus control (pseudoviruses only incubated with cells) and a background control (cells or organoids only without pseudovirus inoculation). A reduction in the luminescence expressed as relative luminescence units (RLUs) in the presence of the serum and mAbs indicated the neutralization efficacy. The 50% inhibitory concentration (IC50) or 90% inhibitory concentration (IC90) was defined as the serum or mAb dilution at which the RLU exhibited a decrease of 50 or 90% in comparison to RLUs of virus control after subtraction of background RLUs. The IC50 and IC90 were calculated using a 4-parameter nonlinear regression analysis in GraphPad Prism 8 (GraphPad Software).

The live virus neutralization assay was conducted in a certified Biosafety Level 3 laboratory. SARS-CoV-2 (0.01 MOI) and SARS-CoV-1 (1 MOI) were mixed with serially diluted mAbs in 96-well plates, followed by a 1-h incubation at 37 °C. The mixtures were then transferred to organoid monolayers in 96-well transwell plates and incubated at 37 °C for 2 h. After rinsing, the infected organoids were incubated with the basal medium in the top and bottom chambers, respectively. At 24 h postincubation, 50 μL of supernatant was collected from each well and mixed with AVL lysis buffer, followed by RNA isolation using the RNeasy Pure mRNA Bead Kit (QIAGEN). The quantification of viral loads was performed with the RT-qPCR assay aforementioned. The percentage reduction in the viral load in antibody-administered organoids compared to that in the organoids without antibody administration indicated the neutralization efficacy. The IC50 and IC90 were calculated using a 4-parameter nonlinear regression analysis in GraphPad Prism 8 (GraphPad Software).

### Statistical Analysis.

Statistical analysis was conducted using GraphPad Prism 8.0. Student’s *t* test or ANOVA test was used to analyze statistical significance as specified in the figure legends. *P ≤ 0.05, **P ≤ 0.01, ***P ≤ 0.001, ****P ≤ 0.0001.

Additional methods are included in *SI Appendix*.

## Supplementary Material

Appendix 01 (PDF)

## Data Availability

All study data are included in the article and/or *SI Appendix*.

## References

[1] D. Planas , Distinct evolution of SARS-CoV-2 omicron XBB and BA.2.86/JN.1 lineages combining increased fitness and antibody evasion. Nat. Commun. **15**, 2254 (2024). 38480689 10.1038/s41467-024-46490-7 PMC10938001

[2] Y. Kaku , Virological characteristics of the SARS-CoV-2 KP.3, LB.1, and KP.2.3 variants. Lancet Infect. Dis. **24**, e482–e483 (2024). 38945150 10.1016/S1473-3099(24)00415-8

[3] B. Ju , Infection with wild-type SARS-CoV-2 elicits broadly neutralizing and protective antibodies against omicron subvariants. Nat. Immunol. **24**, 690–699 (2023). 36914890 10.1038/s41590-023-01449-6 PMC10063446

[4] F. Jian , A generalized framework to identify SARS-CoV-2 broadly neutralizing antibodies. bioRxiv [Preprint] (2024). 10.1101/2024.04.16.589454 (Accessed 16 April 2024).

[5] K. Wang , Memory B cell repertoire from triple vaccinees against diverse SARS-CoV-2 variants. Nature **603**, 919–925 (2022). 35090164 10.1038/s41586-022-04466-x PMC8967717

[6] C. O. Barnes , SARS-CoV-2 neutralizing antibody structures inform therapeutic strategies. Nature **588**, 682–687 (2020). 33045718 10.1038/s41586-020-2852-1 PMC8092461

[7] K. Westendorf , LY-CoV1404 (bebtelovimab) potently neutralizes SARS-CoV-2 variants. Cell Rep. **39**, 110812 (2022). 35568025 10.1016/j.celrep.2022.110812 PMC9035363

[8] Y. Cao , BA.2.12.1, BA.4 and BA.5 escape antibodies elicited by Omicron infection. Nature **608**, 593–602 (2022). 35714668 10.1038/s41586-022-04980-y PMC9385493

[9] A. G. Farrell , Receptor-binding domain (RBD) antibodies contribute more to SARS-CoV-2 neutralization when target cells express high levels of ACE2. Viruses **14**, 2061 (2022). 36146867 10.3390/v14092061 PMC9504593

[10] M. Y. Wu , WHO’s therapeutics and COVID-19 living guideline on mAbs needs to be reassessed. Lancet **400**, 2193–2196 (2022). 10.1016/S0140-6736(22)01938-9 PMC9536776 36209762

[11] M. Hoffmann , Chloroquine does not inhibit infection of human lung cells with SARS-CoV-2. Nature **585**, 588–590 (2020). 32698190 10.1038/s41586-020-2575-3

[12] A. Z. Mykytyn , SARS-CoV-2 Omicron entry is type II transmembrane serine protease-mediated in human airway and intestinal organoid models. J. Virol. **97**, e0085123 (2023). 37555660 10.1128/jvi.00851-23 PMC10506477

[13] D. Pinto , Cross-neutralization of SARS-CoV-2 by a human monoclonal SARS-CoV antibody. Nature **583**, 290–295 (2020). 32422645 10.1038/s41586-020-2349-y

[14] WHO, Therapeutics and COVID-19: Living Guideline (World Health Organization, 2023), 10 November 2023. 35917393

[15] B. Zheng , Comparative effectiveness of sotrovimab and molnupiravir for prevention of severe covid-19 outcomes in patients in the community: Observational cohort study with the OpenSAFELY platform. BMJ **379**, e071932 (2022). 36384890 10.1136/bmj-2022-071932 PMC9667468

[16] M. Drysdale , Real-world effectiveness of sotrovimab for the treatment of SARS-CoV-2 infection during omicron BA.2 subvariant predominance: A systematic literature review. Infection **52**, 1–17 (2024). 37776474 10.1007/s15010-023-02098-5 PMC10811031

[17] M. M. Cheng , Real-world effectiveness of sotrovimab for the early treatment of COVID-19 during SARS-CoV-2 delta and omicron waves in the USA. Infect. Dis. Ther. **12**, 607–621 (2023). 36629998 10.1007/s40121-022-00755-0 PMC9832411

[18] F. A. Lempp , Lectins enhance SARS-CoV-2 infection and influence neutralizing antibodies. Nature **598**, 342–347 (2021). 34464958 10.1038/s41586-021-03925-1

[19] A. Addetia , Neutralization, effector function and immune imprinting of Omicron variants. Nature **621**, 592–601 (2023). 37648855 10.1038/s41586-023-06487-6 PMC10511321

[20] J. B. Case , Resilience of S309 and AZD7442 monoclonal antibody treatments against infection by SARS-CoV-2 Omicron lineage strains. Nat. Commun. **13**, 3824 (2022). 35780162 10.1038/s41467-022-31615-7 PMC9250508

[21] C. Wang , A conserved immunogenic and vulnerable site on the coronavirus spike protein delineated by cross-reactive monoclonal antibodies. Nat. Commun. **12**, 1715 (2021). 33731724 10.1038/s41467-021-21968-w PMC7969777

[22] N. K. Hurlburt , Structural definition of a pan-sarbecovirus neutralizing epitope on the spike S2 subunit. Commun. Biol. **5**, 342 (2022). 35411021 10.1038/s42003-022-03262-7 PMC9001700

[23] D. Pinto , Broad betacoronavirus neutralization by a stem helix-specific human antibody. Science **373**, 1109–1116 (2021). 34344823 10.1126/science.abj3321 PMC9268357

[24] P. Zhou , A human antibody reveals a conserved site on beta-coronavirus spike proteins and confers protection against SARS-CoV-2 infection. Sci. Transl. Med. **14**, eabi9215 (2022). 35133175 10.1126/scitranslmed.abi9215 PMC8939767

[25] E. O. Saphire , Systematic analysis of monoclonal antibodies against Ebola virus GP defines features that contribute to protection. Cell **174**, 938–952.e913 (2018). 30096313 10.1016/j.cell.2018.07.033 PMC6102396

[26] D. R. Burton, Antiviral neutralizing antibodies: From in vitro to in vivo activity. Nat. Rev. Immunol. **23**, 720–734 (2023). 37069260 10.1038/s41577-023-00858-w PMC10108814

[27] J. Zhou , Differentiated human airway organoids to assess infectivity of emerging influenza virus. Proc. Natl. Acad. Sci. U.S.A. **115**, 6822–6827 (2018). 29891677 10.1073/pnas.1806308115 PMC6042130

[28] C. Li , Establishing human lung organoids and proximal differentiation to generate mature airway organoids. J. Vis. Exp. **10**, e63684 (2022). 10.3791/63684 35404361

[29] M. C. Chiu , Apical-out human airway organoids modeling SARS-CoV-2 infection. Viruses **15**, 1166 (2023). 37243252 10.3390/v15051166 PMC10220522

[30] M. C. Chiu , A bipotential organoid model of respiratory epithelium recapitulates high infectivity of SARS-CoV-2 omicron variant. Cell Discov. **8**, 57 (2022). 35710786 10.1038/s41421-022-00422-1 PMC9203776

[31] C. Li , Human airway and nasal organoids reveal escalating replicative fitness of SARS-CoV-2 emerging variants. Proc. Natl. Acad. Sci. U.S.A. **120**, e2300376120 (2023). 37068258 10.1073/pnas.2300376120 PMC10151566

[32] M. C. Chiu , Human nasal organoids model SARS-CoV-2 upper respiratory infection and recapitulate the differential infectivity of emerging variants. mBio **13**, e0194422 (2022). 35938726 10.1128/mbio.01944-22 PMC9426414

[33] D. Wang , SPINK6 inhibits human airway serine proteases and restricts influenza virus activation. EMBO Mol. Med. **14**, e14485 (2022). 34826211 10.15252/emmm.202114485 PMC9976594

[34] B. Ju , Human neutralizing antibodies elicited by SARS-CoV-2 infection. Nature **584**, 115–119 (2020). 32454513 10.1038/s41586-020-2380-z

[35] L. de Campos-Mata , A monoclonal antibody targeting a large surface of the receptor binding motif shows pan-neutralizing SARS-CoV-2 activity. Nat. Commun. **15**, 1051 (2024). 38316751 10.1038/s41467-024-45171-9 PMC10844294

[36] T. N. Starr , SARS-CoV-2 RBD antibodies that maximize breadth and resistance to escape. Nature **597**, 97–102 (2021). 34261126 10.1038/s41586-021-03807-6 PMC9282883

[37] W. Sungnak , SARS-CoV-2 entry factors are highly expressed in nasal epithelial cells together with innate immune genes. Nat. Med. **26**, 681–687 (2020). 32327758 10.1038/s41591-020-0868-6 PMC8637938

[38] F. Hikmet , The protein expression profile of ACE2 in human tissues. Mol. Syst. Biol. **16**, e9610 (2020). 32715618 10.15252/msb.20209610 PMC7383091

[39] S. Ramirez , Overcoming culture restriction for SARS-CoV-2 in human cells facilitates the screening of compounds inhibiting viral replication. Antimicrob. Agents Chemother. **65**, e0009721 (2021). 33903110 10.1128/AAC.00097-21 PMC8406809

[40] M. P. O’Brien , Subcutaneous REGEN-COV antibody combination to prevent Covid-19. N. Engl. J. Med. **385**, 1184–1195 (2021). 34347950 10.1056/NEJMoa2109682 PMC8362593

[41] D. Yamasoba , Neutralisation sensitivity of SARS-CoV-2 omicron subvariants to therapeutic monoclonal antibodies. Lancet Infect. Dis. **22**, 942–943 (2022). 35690075 10.1016/S1473-3099(22)00365-6 PMC9179126

[42] H. Montgomery , Efficacy and safety of intramuscular administration of tixagevimab-cilgavimab for early outpatient treatment of COVID-19 (TACKLE): A phase 3, randomised, double-blind, placebo-controlled trial. Lancet Respir. Med. **10**, 985–996 (2022). 35688164 10.1016/S2213-2600(22)00180-1 PMC9173721

[43] J. Baggen, E. Vanstreels, S. Jansen, D. Daelemans, Cellular host factors for SARS-CoV-2 infection. Nat. Microbiol. **6**, 1219–1232 (2021). 34471255 10.1038/s41564-021-00958-0

[44] T. P. Peacock , The furin cleavage site in the SARS-CoV-2 spike protein is required for transmission in ferrets. Nat. Microbiol. **6**, 899–909 (2021). 33907312 10.1038/s41564-021-00908-w PMC7619196

[45] A. Z. Mykytyn , SARS-CoV-2 entry into human airway organoids is serine protease-mediated and facilitated by the multibasic cleavage site. Elife **10**, e64508 (2021). 33393462 10.7554/eLife.64508 PMC7806259

[46] M. Hoffmann , SARS-CoV-2 cell entry depends on ACE2 and TMPRSS2 and is blocked by a clinically proven protease inhibitor. Cell **181**, 271–280.e278 (2020). 32142651 10.1016/j.cell.2020.02.052 PMC7102627

[47] A. Shulla , A transmembrane serine protease is linked to the severe acute respiratory syndrome coronavirus receptor and activates virus entry. J. Virol. **85**, 873–882 (2011). 21068237 10.1128/JVI.02062-10 PMC3020023

[48] N. Iwata-Yoshikawa , TMPRSS2 contributes to virus spread and immunopathology in the airways of murine models after coronavirus infection. J. Virol. **93**, e01815-18 (2019). 30626688 10.1128/JVI.01815-18 PMC6401451

[49] Y. Zhang , Transmembrane serine protease TMPRSS2 implicated in SARS-CoV-2 infection is autoactivated intracellularly and requires N-glycosylation for regulation. J. Biol. Chem. **298**, 102643 (2022). 36309092 10.1016/j.jbc.2022.102643 PMC9598255

[50] S. Luo , An antibody from single human V-rearranging mouse neutralizes all SARS-CoV-2 variants through BA.5 by inhibiting membrane fusion. Sci. Immunol. **7**, eadd5446 (2022). 35951767 10.1126/sciimmunol.add5446 PMC9407951

[51] H. Sun , Structural basis for broad neutralization of human antibody against Omicron sublineages and evasion by XBB variant. J. Virol. **97**, e0113723 (2023). 37855619 10.1128/jvi.01137-23 PMC10688377

