# Supplementary material for: Organoid-based neutralization assays reveal a distinctive profile of SARS-CoV-2 antibodies and recapitulate the real-world efficacy
Source: Proc Natl Acad Sci U S A. 2025 Aug 28;122(35):e2509616122. doi: 10.1073/pnas.2509616122 (PMC12415223; doi:10.1073/pnas.2509616122)
Supplement: Supplementary file 1 — Appendix 01 (PDF) [file pnas.2509616122.sapp.pdf]

PNAS

Supporting Information for

Organoid-based neutralization assays reveal a distinctive profile of SARS-CoV-2 antibodies and recapitulate the real-world efficacy

Zhixin Wan[a,1], Cun Li[a,1], Ying Zhou[a], Yifei Yu[a], Man Chun Chiu[a,b], Jingjing Huang[a,b], Shuxin Zhang[a], Xiaoxin Zhu[a,b], Qiaoshuai Lan[a,b], Yanlin Deng[a,b], Wei Xue[a], Chengfan Jiang[a], Jiali Wu[a], Zijun Zhao[a], Jian-Piao Cai[a], Lin Huang[c], Yong Zhang[c], Xiaojuan Liu[d], Zheng Zhang[e], Hin Chu[a,b,f,h], Linqi Zhang[g,h], Zhiwei Chen[a,b,f,h], Kelvin Kai-Wang To[a,b,f,h], Kwok Yung Yuen[a,b,f,h], Hans Clevers[i,j,2], Jie Zhou[a,b,c,f,h,2]

Affiliations:

[a]Department of Microbiology, Li Ka Shing Faculty of Medicine, The University of Hong Kong, Pokfulam, Hong Kong, China

[b]Centre for Virology, Vaccinology and Therapeutics, Hong Kong Science and Technology Park, Hong Kong, China

[c]BiomOrgan Ltd, Hong Kong, China

[d]Clinical Stem Cell Research Center, Peking University Third Hospital, Beijing 100191, China

[e]Institute for Hepatology, National Clinical Research Center for Infectious Disease, Shenzhen Third People's Hospital, Shenzhen 518112, China

[f]State Key Laboratory of Emerging Infectious Diseases, The University of Hong Kong, Hong Kong, China

[g]Center for Global Health and Infectious Diseases, Comprehensive AIDS Research Center and Beijing Advanced Innovation Center for Structural Biology, School of Medicine, and Vanke School of Public Health, Tsinghua University, Beijing 100084, China

[h]Pandemic Research Alliance Unit at the University of Hong Kong

[i]Oncode Institute, Hubrecht Institute, Royal Netherlands Academy of Arts and Sciences, and University Medical Center Utrecht, Utrecht 3584 CT, the Netherlands

[j]Roche Pharmaceutical Research and Early Development, Basel CH-4070, Switzerland

[1]These authors contributed equally.

Correspondence to: Hans Clevers, Email: h.clevers@hubrecht.eu Pharma, Research and Early Development of F. Hoffmann-La Roche Ltd, CH-4070 Basel, Switzerland, and Jie Zhou, Email: jiezhou@hku.hk Department of Microbiology, The University of Hong Kong, 102 Pokfulam Road, Hong Kong, China

This PDF file includes:

Supporting methods
Figures S1 to S9
SI References

Supporting methods

Cell lines, virus infection, and detection

Vero E6-TMPRSS2 cells were maintained in Dulbecco's Modified Eagle Medium (DMEM; Gibco), supplemented with 10% Fetal Bovine Serum (FBS; Gibco), 1% penicillin-streptomycin (P/S; Gibco), and 1 mg/ml of Geneticin Selective Antibiotic (G418; Gibco), at 37°C with 5% CO_2 in a humidified incubator. Huh-7, Vero E6, and 293T-ACE2 cell lines were maintained in DMEM medium with 10% FBS and 1% P/S. The 293T-ACE2 cells were maintained in the same medium, additionally supplemented with 100 μg/ml Hygromycin B. All cell lines were maintained at 37°C in a 5% CO_2 incubator. Upon reaching approximately 90% confluence, the cells were disassociated using EDTA-trypsin (Gibco) and passaged with a ratio of 1:6. Regular mycoplasma screening was conducted to ensure that all cultures remained free from mycoplasma contamination.

SARS-CoV-2 isolate HKU-001a (wild type, GenBank accession number MT230904), the Omicron subvariant BA.5.2 (GISAID accession no. EPI_ISL_13777658), EG.5.1 (GISAID accession number: EPL_ISL_18461518), and SARS-CoV-1 (GZ50 strain; GenBank accession no. AY304495) were plaque-purified and subsequently propagated in Vero E6 cells. Viral titers were determined using plaque assays as described previously (1). After rinsing with a basal medium, which is Advanced DMEM/F-12 (Gibco) supplemented with 1% HEPES, 1% GlutaMAX, and 1% Penicillin/Streptomycin, nasal organoids were inoculated with the indicated viruses at specified multiplicities of infection (MOIs) for 2 hours at 37°C, the infected nasal organoids were then incubated in the basal medium. Cell-free culture media were harvested at the indicated time points post-infection to detect viral replication kinetics. RNA extraction was performed using the MiniBEST Viral RNA/DNA Extraction Kit (Takara); and viral loads were quantified via RT-qPCR assays targeting the viral RNA-dependent RNA polymerase (RdRp) gene, utilizing the QuantiNova Probe RT-PCR Kit (Qiagen, 208354). Viral titration was conducted using $TCID_{50}$ assays as reported previously (2, 3). All experiments involving live viruses were performed in the biosafety level 3 laboratory.

Immunofluorescence staining, confocal imaging, and flow cytometry

The nasal organoids were applied to immunofluorescence staining to characterize the cellular composition as described elsewhere (3-7). After fixation with 4% paraformaldehyde (PFA), virus- and mock-infected organoids were applied to immunostaining and confocal imaging as described previously (1). In brief, the fixed organoid monolayers were permeabilized with 0.1% Triton X-100 for 10 minutes, followed by a 1-hour blocking with a protein block (Dako). The monolayers were then incubated with primary antibodies, followed by secondary antibodies. Cell nuclei and actin filaments were counterstained with DAPI (Thermo Fisher) and Phalloidin-Atto 647 (Sigma-Aldrich), respectively. Subsequently, the organoids were whole-mounted using ProLong™ Glass Antifade Mountant (Invitrogen). Confocal images were captured using a Carl Zeiss LSM 980 confocal microscope, and image processing was conducted with ZEN blue software.

For flow cytometry analysis, the organoids were dissociated into single cells using 10-20 mM EDTA (Invitrogen) at 37°C, followed by fixation with 4% PFA, permeabilization with 0.1% Triton X-100, and immunostaining with primary and secondary antibodies. Cells stained with isotype antibodies served as controls for gating the positive population. The immune-labeled cells were resuspended in 2% FBS/PBS solution and analyzed using the NovoCyte Advanteon BVYG analyzer. Data interpretation was performed using FlowJo software, ensuring a thorough analysis of the immunostaining results.

Western blot

The cell lines and nasal organoids were lysed using radioimmunoprecipitation assay (RIPA) buffer (89901, Thermo Fisher Scientific) supplemented with a protease inhibitor (4693159001, Roche). The lysates were then separated via 10% SDS-polyacrylamide gel electrophoresis (SDS-PAGE) and subsequently transferred to a 0.22 μm polyvinylidene difluoride (PVDF) membrane (Bio-Rad). Subsequently, the membranes were blocked and incubated with specific primary antibodies at 4°C overnight, followed by incubation with horseradish peroxidase (HRP)-conjugated secondary antibodies (Thermo Fisher Scientific) (8, 9). The protein signals were developed using the

Immobilon Crescendo Western HRP Substrate (WBLUR0500, Merck Millipore) and visualized with the Alliance Imager apparatus (Uvitec, Cambridge UK). β-Actin was detected using a mouse anti-human β-actin antibody (MAB8929, R&D Systems) as a loading control.

Pseudovirus infectivity assay

Lenti-X 293T cells (TAKARA, 632180) were cultured in DMEM supplemented with 10% FBS and 1% P/S. SARS-CoV-1 and SARS-CoV-2 variant pseudoviruses were produced through co-transfecting a human immunodeficiency virus (HIV) backbone reporter plasmid (pNL4-3-R-E-luciferase) and pcDNA3.1 vector (Invitrogen) encoding the respective spike proteins into Lenti-X 293T cells (Takara Bio). After a 48 ~72-hour incubation, supernatants were harvested and centrifuged at 800 *g* for 5 minutes to eliminate cell debris after filtration through a 0.45 μm membrane. The viral titer was quantified by detecting luciferase activity with the Bright-Glo Luciferase Assay System (Promega), and controls were transfected with equal amounts of empty vector instead of the named plasmid.

To evaluate the entry efficiencies of pseudoviruses, we inoculated nasal organoids or the cell lines with the pseudoviruses side by side. After 72-hour incubation, the organoids and cells were subjected to luciferase assays employing the Bright-Glo Luciferase assay system (Promega, E2620). In the experiments of protease inhibition, monolayers were pretreated with either 50 μM Camostat (Sigma-Aldrich), 50 μM E64D (Sigma-Aldrich), or DMSO, followed by pseudovirus transduction, and luciferase assay at 72 hours post-incubation.

Imaging analysis of neutralization activity

For imaging analysis of neutralization activity, the infection and administration of mAb were conducted as aforementioned. The infected organoids, after fixation with 4% PFA for 1 hour, permeabilization with 0.1% Triton-X and blockage with 100 μl of 3% bovine serum albumin (BSA), were stained using a homemade antibody targeting the SARS-CoV-2 nucleocapsid protein (NP) and a secondary antibody. Nuclei were counterstained with DAPI (Thermo Fisher Scientific). Subsequently, the 96-well transwell plate was scanned using the IN Cell Analyzer 6500HS, according to the manufacturer's guidelines. All acquired images were analyzed with IN Carta software to quantify the percentage of infected cells.

Source of monoclonal antibodies

The mAbs studied in this paper, VIR-7831 (HY-P99340), AZD8895 (HY-P99556), BRII-196 (HY-P99435), REGN10933 (HY-P99341), AZD1061 (HY-P99604), LY-CoV1404 (HY-P99103), REGN10987 (HY-P99342), DXP-604 (PTXCOV-A581), S2E12 (PTXCOV-A579), and S2K146 (PTXCOV-A582) were purchased from MCE and Prote Genix. XGv-347, LY-CoV555, XGv-387, BD55-3152, BD-692, C581, XGv-293, CC40.8, S2P6, and CV3-25 were kindly provided by Prof Yunlong Cao and Prof Zheng Zhang and have been described previously.

Source of Convalescent Sera

Peripheral blood was collected from a volunteer who had received 3 doses of Sinovac inactivated vaccine against a SARS-CoV-2 ancestral strain and 1 dose of mRNA-based BNT162b2 against an ancestral spike. Sera were isolated from centrifuged blood samples and then stored at -80°C.

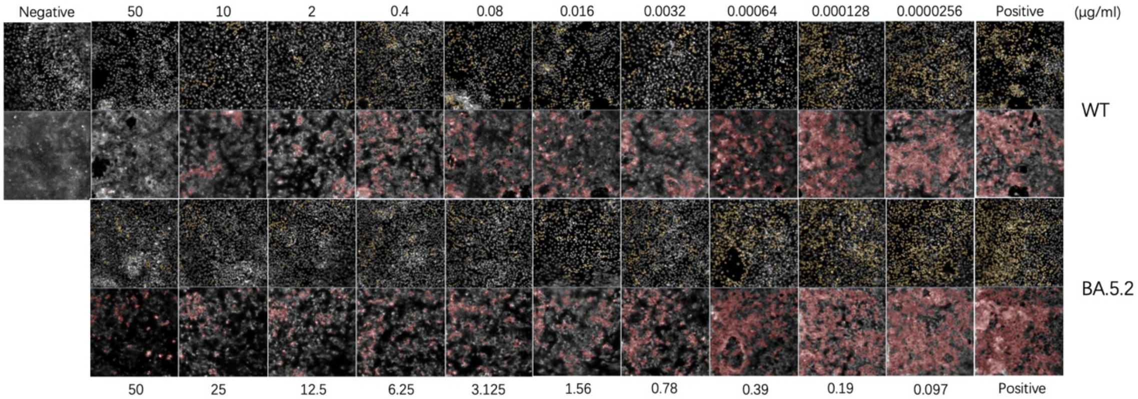

Fig. S1. Image-based analysis for assessing mAb in nasal organoids. Serial dilution of VIR-7831 was tested against live SARS-CoV-1 (1 MOI) or SARS-CoV-2 BA.5.2 (0.01 MOI) infection in nasal organoids. After 24 hpi, staining with viral NP antibody, representative images of infected cells at different mAb concentration treatments after processing with IN Carta software, NP (red), cell nucleus (yellow), n = 4.

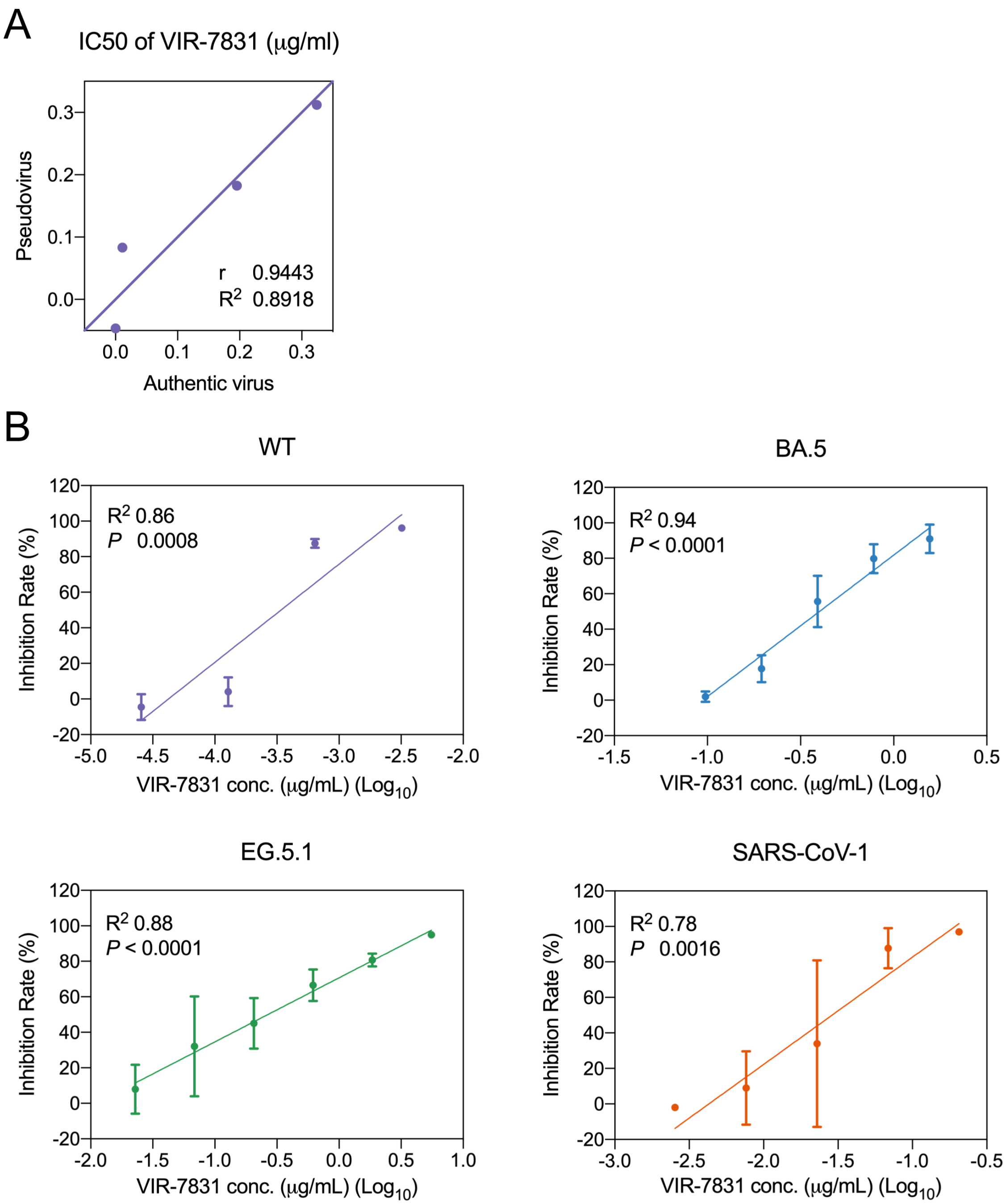

Fig. S2. Correlation and agreement between pseudovirus and authentic virus neutralization activity in nasal organoids. (A) The correlation of VIR-7831 neutralization activity (IC50) measured in pseudovirus and live virus, assessed using the Pearson correlation coefficient. (B) The neutralization potency of VIR-7831 against the indicative live virus and pseudovirus was tested in nasal organoids. A simple linear regression analysis was used to assess the correlation between inhibition and dilution factors.

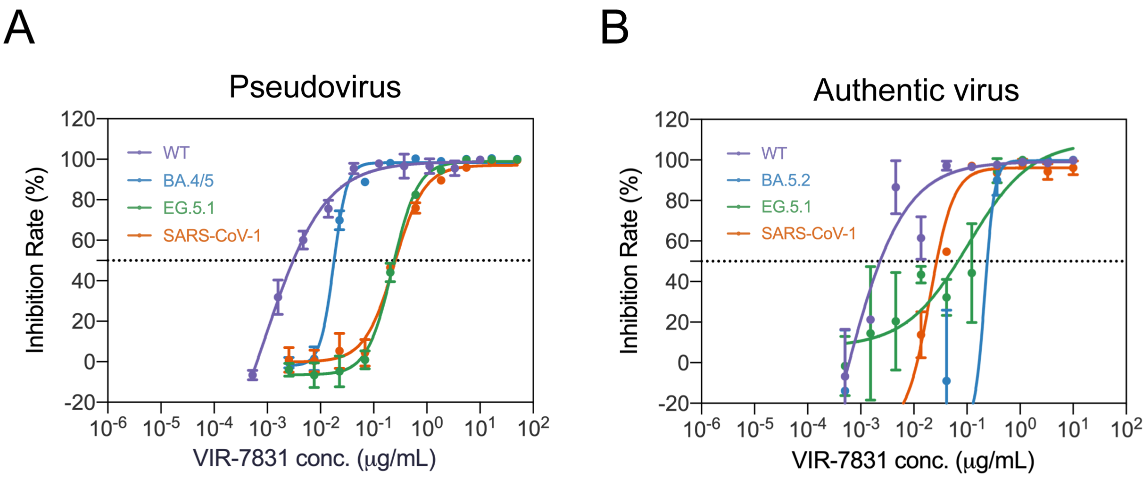

C

	IC50 (ng/mL)				IC90 (ng/mL)			
	WT	BA.5	EG.5.1	SARS-CoV-1	WT	BA.5	EG.5.1	SARS-CoV-1
Pseudovirus	0.857	226	246	17.7	15.0	754	1017	33.4
Live virus	0.439	217	106	20.1	7.6	346	2097	61.4

Fig. S3. Neutralization activity of VIR-7831 in nasal organoids derived from a different donor. The neutralization activity of VIR-7831 against SARS-CoV-1 and SARS-CoV-2 variants using (A) pseudoviruses or (B) authentic virus in another donor of nasal organoids, indicated by (C) IC50 (ng/mL). Data are mean ± SD and n = 4.

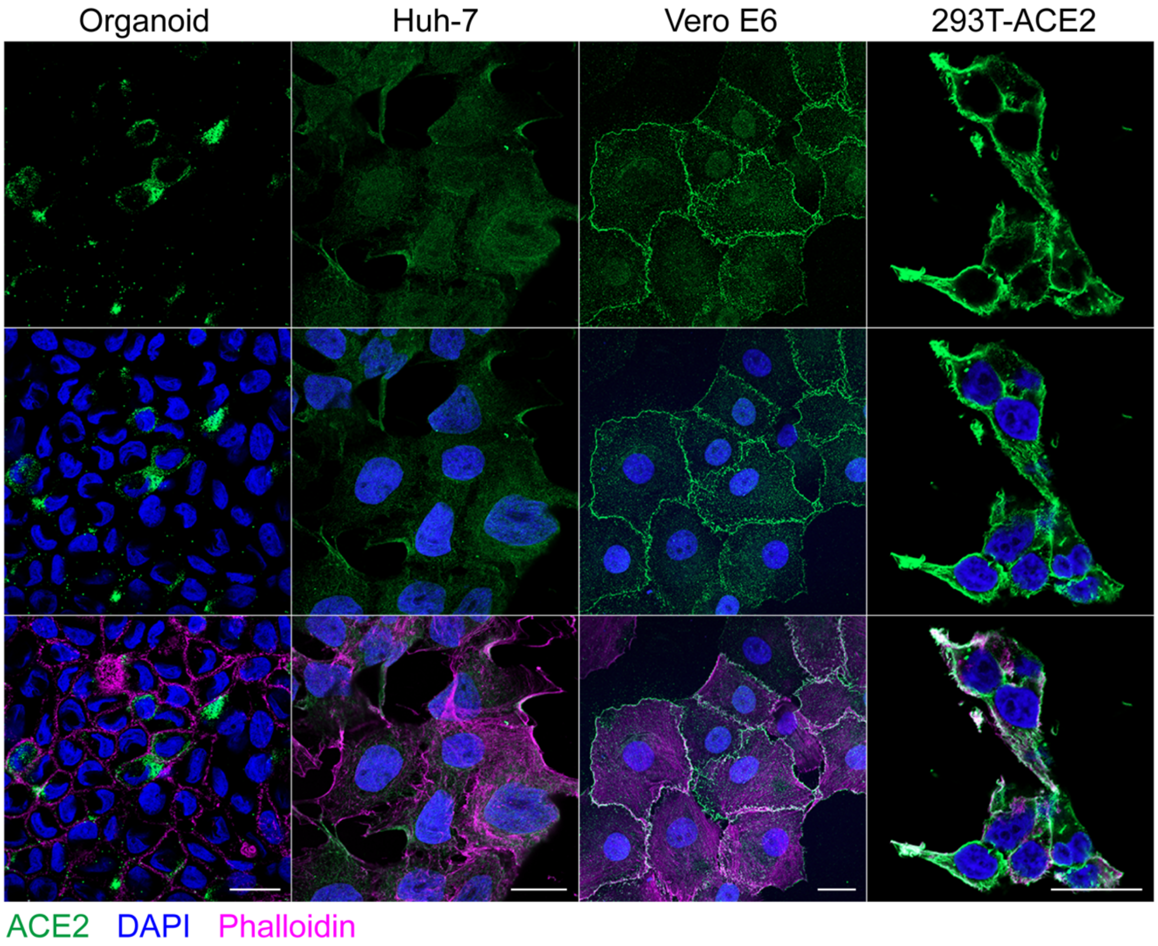

Fig. S4. ACE2 expression in nasal organoids and different cell lines. Immunofluorescent images of ACE2 expression in nasal organoids and the indicated cell lines. ACE2 (green), DAPI (blue), and Phalloidin-647 (Purple). Scale bar, 20 μm.

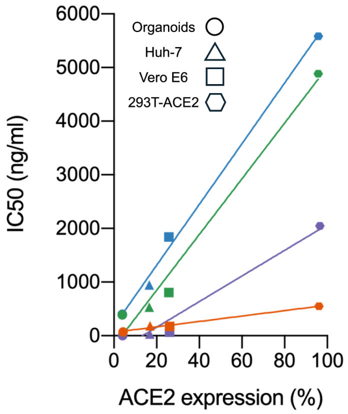

	R^2	*P*
WT	0.9633	0.0185
BA.4/5	0.9957	0.0021
EG.5.1	0.9793	0.0104
SARS-CoV-1	0.9861	0.0070

Fig. S5. Correlation between the percentage of ACE2$^+$ cells as detected by flow cytometry and IC50 values in nasal organoids and indicated cell lines. Nonparametric, two-tailed Spearman correlation was performed using GraphPad Prism.

A

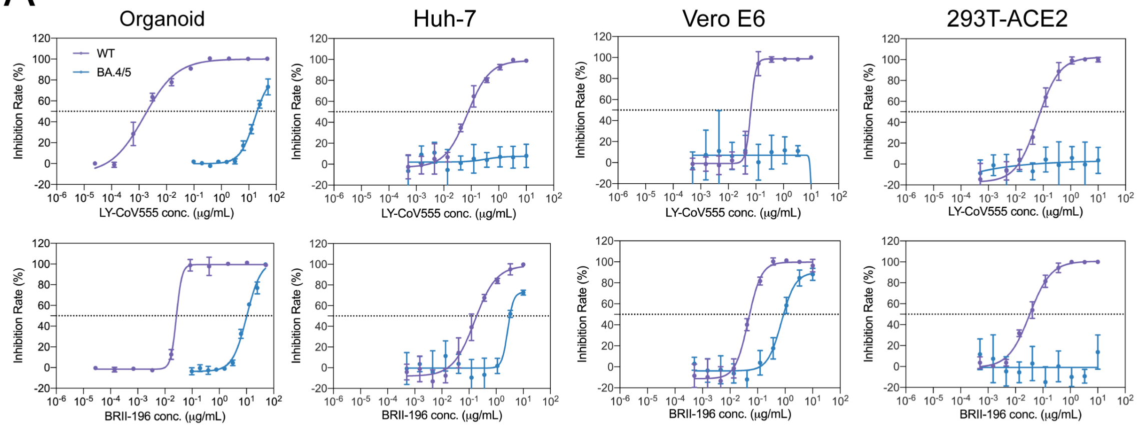

B

mAb	Pseudovirus	IC50 (ng/mL)			
		Organoid	Huh-7	Vero E6	293T-ACE2
LY-CoV555	WT	1.602	72.4	65.02	62.18
	BA.4/5	16100	>10000	>10000	>10000
BRII-196	WT	24.64	161.8	45.92	33.06
	BA.4/5	10220	2666	740	>10000

Fig. S6. LY-CoV555 and BRII-196 neutralization efficacy in nasal organoids and different cell lines. (A) Neutralization activity of LY-CoV555 and BRII-196 against SARS-CoV-2 WT and BA.4/5 pseudovirus in nasal organoids and indicated cell lines. Data show the mean ± SD (n = 4). (B) The heatmap shows the IC50 and IC90 values (ng/mL) of LY-CoV555 and BRII-196 against the indicated pseudoviruses. The neutralizing potency is red-white-blue color-coded, with red being the strongest neutralization of each mAb to the indicated pseudoviruses among the test models.

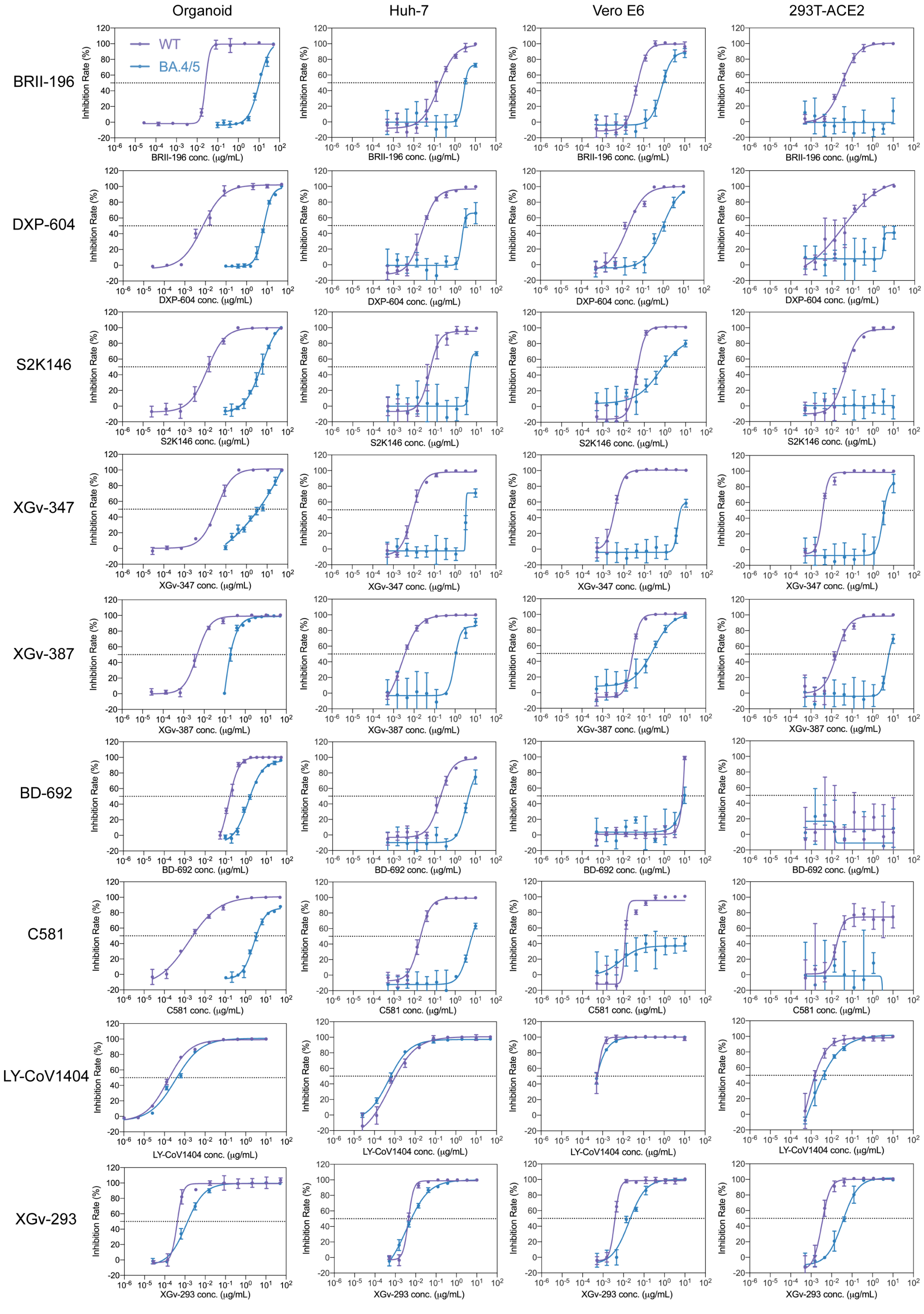

Fig. S7. The efficacy of RBD targeting mAbs in organoids and different cell lines. Neutralization curves of selected class 1/2 and class 3 mAbs against SARS-CoV-2 WT and BA.4/5 pseudovirus, related to **Fig. 4i**. The horizontal dotted lines on each graph indicate 50% and 0% neutralization. Data are mean ± SD and n = 4.

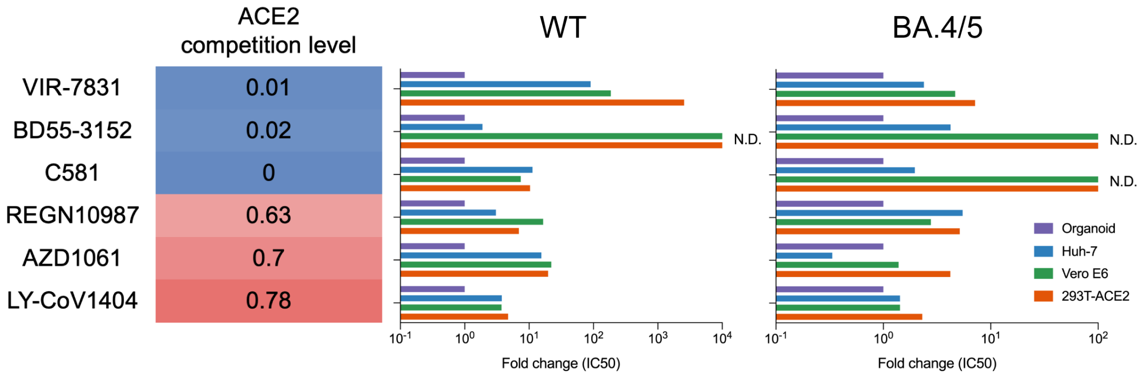

Fig. S8. ACE2-competition level and neutralization activity of class 3 mAbs in organoids and different cell lines. The ACE2-competition level is determined by competition ELISA, with high competition colored red and low competition colored blue. The fold changes in neutralization IC50 values of 6 class 3 mAbs against SARS-CoV-2 WT and BA.4/5 pseudoviruses among different cell lines compared with organoid.

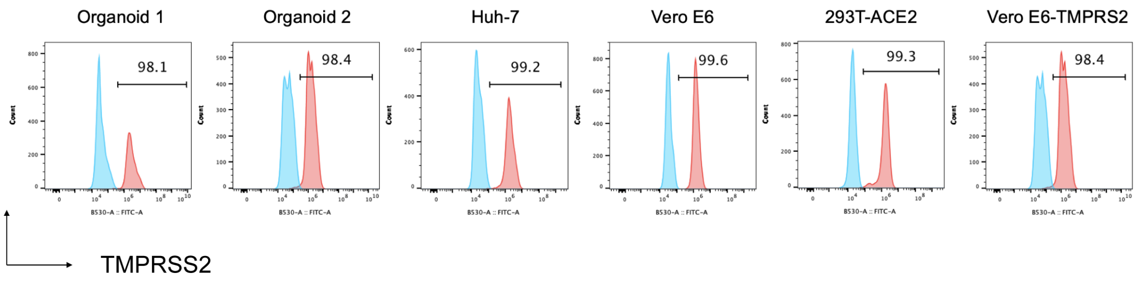

Fig. S9. TMPRSS2 expression in different models. Representative histograms show the percentage of TMPRSS2+ cells (red) in the organoids and cell lines. Isotype control (blue). Related to **Fig. 5g**.

SI References

1. X. Zhao *et al.*, Human Intestinal Organoids Recapitulate Enteric Infections of Enterovirus and Coronavirus. *Stem Cell Reports* **16**, 493-504 (2021).
2. C. Li *et al.*, Human airway and nasal organoids reveal escalating replicative fitness of SARS-CoV-2 emerging variants. *Proc Natl Acad Sci U S A* **120**, e2300376120 (2023).
3. J. Zhou *et al.*, Infection of bat and human intestinal organoids by SARS-CoV-2. *Nat Med* **26**, 1077-1083 (2020).
4. M. C. Chiu *et al.*, Apical-Out Human Airway Organoids Modeling SARS-CoV-2 Infection. *Viruses* **15** (2023).
5. D. Wang *et al.*, SPINK6 inhibits human airway serine proteases and restricts influenza virus activation. *EMBO Mol Med* **14**, e14485 (2022).
6. J. Zhou *et al.*, Active replication of Middle East respiratory syndrome coronavirus and aberrant induction of inflammatory cytokines and chemokines in human macrophages: implications for pathogenesis. *J Infect Dis* **209**, 1331-1342 (2014).
7. J. Zhou *et al.*, Human intestinal tract serves as an alternative infection route for Middle East respiratory syndrome coronavirus. *Science advances* **3**, eaao4966 (2017).
8. J. Zhou *et al.*, Kruppel-like factor 15 activates hepatitis B virus gene expression and replication. *Hepatology* **54**, 109-121 (2011).
9. J. Zhou *et al.*, Identification and characterization of GLDC as host susceptibility gene to severe influenza. *EMBO Mol Med* **11** (2019).
